# WASF2 Serves as a Potential Biomarker and Therapeutic Target in Ovarian Cancer: A Pan-Cancer Analysis

**DOI:** 10.3389/fonc.2022.840038

**Published:** 2022-03-14

**Authors:** Xiaofeng Yang, Yuzhen Ding, Lu Sun, Meiting Shi, Ping Zhang, Andong He, Xiaotan Zhang, Zhengrui Huang, Ruiman Li

**Affiliations:** ^1^ Department of Obstetrics and Gynecology, The First Affiliated Hospital of Jinan University, Guangzhou, China; ^2^ Department of Pathology, The First Affiliated Hospital of Jinan University, Guangzhou, China

**Keywords:** pan-cancer, WASF2, biomarker, immune, therapeutic

## Abstract

**Background:**

Wiskott-Aldrich syndrome protein family member 2 (WASF2) has been shown to play an important role in many types of cancer. Therefore, it is worthwhile to further study expression profile of WASF2 in human cancer, which provides new molecular clues about the pathogenesis of ovarian cancer.

**Methods:**

We used a series of bioinformatics methods to comprehensively analyze the relationship between WASF2 and prognosis, tumor microenvironment (TME), immune infiltration, tumor mutational burden (TMB), microsatellite instability (MSI), and tried to find the potential biological processes of WASF2 in ovarian cancer. Biological behaviors of ovarian cancer cells were investigated through CCK8 assay, scratch test and transwell assay. We also compared WASF2 expression between epithelial ovarian cancer tissues and normal ovarian tissues by using immunohistochemical staining.

**Results:**

In the present study, we found that WASF2 was abnormally expressed across the diverse cancer and significantly correlated with overall survival (OS) and progression-free interval (PFI). More importantly, the WASF2 expression level also significantly related to the TME. Our results also showed that the expression of WASF2 was closely related to immune infiltration and immune-related genes. In addition, WASF2 expression was associated with TMB, MSI, and antitumor drugs sensitivity across various cancer types. Functional bioinformatics analysis demonstrated that the WASF2 might be involved in several signaling pathways and biological processes of ovarian cancer. A risk factor model was found to be predictive for OS in ovarian cancer based on the expression of WASF2. Moreover, *in vitro* experiments, it was demonstrated that the proliferative, migratory and invasive capacity of ovarian cancer cells was significantly inhibited due to WASF2 knockdown. Finally, the immunohistochemistry data confirmed that WASF2 were highly expressed in ovarian cancer.

**Conclusions:**

Our study demonstrated that WASF2 expression was associated with a poor prognosis and may be involved in the development of ovarian cancer, which might be explored as a potential prognostic marker and new targeted treatments.

## Introduction

Malignant neoplasms are one of the leading causes of mortality and disability worldwide and there are still no effective treatments for patients with late stages of these diseases (1–3). Currently, surgery, radiotherapy, and chemotherapy remain to be the main modalities for the treatment of cancer (4, 5). In recent years, cancer immunotherapy has become the major theme, whose safety and efficacy have been gradually recognized (6, 7). With the extensive using of genome sequencing technology and public databases, it is possible to identify potential biomarkers and therapeutic target by using pan-cancer analysis with genes expression.

The morphology and cytoskeleton of migrating cell are induced by rearrangement of the actin cytoskeleton at the leading and trailing edges of cells and are accompanied by the formation of lamellipodia and filopodia (8). Wiskott-Aldrich syndrome protein family member 2 (WASF2) is essential for lamellipodium formation and mediates cell migration and invasion (9). The gene product is a protein that forms a multiprotein complex that links receptor kinases and actin. WASF2 has a C-terminal verprolin homology domain which can bind and activate the actin-nucleating actin-related protein-2/3 (Arp2/3) complex (10, 11). The multiprotein complex serves to a central biochemical mechanism in transduce signals that involve changes in cell shape, motility or function. Recently, some evidence has suggested that WASF2 is overexpressed in some of types of cancers and the high expression is correlated with metastasis, poor prognosis, and resistance to treatment (12–16).

The tumor microenvironment (TME) refers to the “ecological niche” surrounding the tumor, which is composed of multiple cell types, supportive matrix and soluble factors (17). TME contains a complex immune cell microenvironment, including innate immune response cells, such as natural killer (NK) cells, macrophages and dendritic cells; these cells also play roles in adaptive immune responses, such as CD8^+^ and CD4^+^ T cells (18). Studies have shown that WASF2 is closely related to the tumor immune microenvironment (19, 20). Conditional gene knockout of WASF2 in T cells can lead to severe autoimmunity (21). Therefore, WASF2 may be involved in the regulation of tumor immune microenvironment.

Nevertheless, most research studies on the roles of WASF2 in tumors were focused on individual cancer types and the sample sizes were often limited. There has been so far no pan-cancer studies focusing on the association between WASF2 and various cancers. Therefore, multi-omics pan-cancer analysis of the WASF2 can not only help to discover common phenotypic characteristics of tumors, but also carry out an in-depth interpretation about the causes of key molecular events and their own internal regulatory mechanisms. In this study, we comprehensively analyzed the expression level of WASF2 and their relationship with prognosis in different types of malignancy from multiple public databases, such as TCGA, Genotype Tissue-Expression (GTEx), and Cancer Cell Line Encyclopedia (CCLE). We also explored the associations between WASF2 expression and immune infiltration levels, drug sensitivity, and tumor mutational burden (TMB) across 33 types of cancer. Gene set enrichment analysis (GSEA) were also applied to explore potential mechanisms. Finally, we constructed a prediction model based on the expression of WASF2 and clinical symptoms to predict the prognosis of ovarian cancer and confirmed that the high expression of WASF2 in ovarian cancer. In addition, we found that the silence of WASF2 could inhibit the proliferation, migration and invasion of ovarian cancer cells.

Our studies revealed the possible roles of WASF2 across cancer, demonstrating that WASF2 would be a potential candidate for clinical biomarker, which could be used as a potential diagnostic and prognostic biomarker in many cancers, especially in ovarian cancer. Meanwhile, this study provided a novel perspective on the roles of WASF2 in tumor immunotherapy.

## Materials and Methods

### Sample Information and WASF2 Expression Analysis in Human Pan-Cancer

The TCGA (https://portal.gdc.cancer.gov/) is currently the largest cancer genetic information database, which stores data including gene expression profiles, copy number variation (CNV), and single nucleotide polymorphism (SNP). The mRNA expression profile data and SNP data of 33 cancer types were downloaded from the TCGA database for subsequent analysis. The difference expression level of WASF2 between cancer and normal tissues was analyzed with a combination of the normal tissues data from the GTEx database (https://commonfund.nih.gov/GTEx) and the TCGA. The online database UALCAN (ualcan.path.uab.edu/) was used to obtain the differential expression levels of WASF2 in ovarian cancer and normal ovarian tissues. Gene-centric RMA-normalized mRNA expression data for each tumor cell line was downloaded from the CCLE database (https://portals.broadinstitute.org/ccle). The expression data of WASF2 in different normal cells were obtained through the HPA database (https://www.proteinatlas.org/humanproteome/pathology). The 33 TCGA cancer types included adrenocortical carcinoma (ACC), bladder urothelial carcinoma (BLCA), breast invasive carcinoma (BRCA), cervical squamous cell carcinoma (CESC), cholangiocarcinoma (CHOL), colon adenocarcinoma (COAD), lymphoid neoplasm diffuse large B cell lymphoma (DLBC), esophageal carcinoma (ESCA), glioblastoma multiforme (GBM), brain lower grade glioma (LGG), head and neck squamous cell carcinoma (HNSC), kidney chromophobe (KICH), kidney renal clear cell carcinoma (KIRC), kidney renal papillary cell carcinoma (KIRP), acute myeloid leukemia (LAML), liver hepatocellular carcinoma (LIHC), lung adenocarcinoma (LUAD), lung squamous cell carcinoma (LUSC), mesothelioma (MESO), ovarian serous cystadenocarcinoma (OV), pancreatic adenocarcinoma (PAAD), pheochromocytoma and paraganglioma (PCPG), prostate adenocarcinoma (PRAD), rectum adenocarcinoma (READ), sarcoma (SARC), skin cutaneous melanoma (SKCM), stomach adenocarcinoma (STAD), testicular germ cell tumors (TGCT), thyroid carcinoma (THCA), thymoma (THYM), uterine corpus endometrial carcinoma (UCEC), uterine carcinosarcoma (UCS), and uveal melanoma (UVM). All expression data were normalized log transformed prior to analysis. Moreover, tumor samples were divided into subgroups based on the tumor stage to evaluate correlations between gene expression levels and tumor stage or grade.

### Survival and Prognosis Analysis

Survival information were extracted from the Xena database to further analyze the relationship between gene expression and patient prognosis of TCGA. Two major endpoints, overall survival (OS) and progression-free interval (PFI), were selected as the main indicators of prognostic assessment. OS refers to the duration from the date of diagnosis to the date of death. PFI is defined as the time from the date of diagnosis until the date of the first occurrence of a novel tumor event, which includes new primary tumor, distant metastasis, local recurrence, the progression of the disease or death due to cancer. Based on the median risk score, patients were divided into high (≥ median) and low (< median) risk groups. Survival analysis was estimated by the Kaplan-Meier (KM) method, and any differences between survival curves were compared using the log-rank test. The survival curves were delineated according to the high and low-risk value using the R packages “survival” and “survminer”. In addition, Cox analysis was used to explore the relationship between WASF2 expression and prognosis. The forest plot was used to display the *P* value, hazard ratios (HR) and 95% confidence interval of each cancer for the effect of gene expression on OS and PFI using the R package “forestplot”.

### Immune Cell Infiltration Analysis

CIBERSORT algorithm was used to calculate the relative proportion of infiltrating immune cells and analyze the correlation between gene expression and immune cell content. Patient samples were divided into two groups according to the median expression of WASF2 gene (high vs. low expression). Differences between groups were compared by Wilcox test through R software. TIMER database was used to calculate the immune cell infiltration information of each tumor. The heat map could be used to show the relationship between the target gene in pan-cancer and immune invasion. Positive correlations were displayed in red and negative correlations in blue (Using ggplot2, ggpubr, patchwork, showtext package for visualization). Furthermore, the TISIDB website explored the potential relationship between WASF2 expression and immune regulatory factors (chemokines, immune checkpoint, immunosuppressive agents, immunostimulatory factors, MHC molecules, etc.). Finally, the relationship between WASF2 and common tumor-related regulatory genes (autophagy, DNA repair, ferroptosis, hypoxia, pyroptosis, and TGF-β signaling gene) were also analyzed.

### Tumor Mutation Burden Analysis

Tumor mutation burden (TMB) is defined as the total number of somatic gene coding errors, base substitutions, insertions or deletions detected per million bases. In this study, TMB was calculated using the mutation frequency and the number of mutations/exon length of each tumor sample, and dividing the non-synonymous mutation site by the total length of the protein coding region. The microsatellite instability (MSI) value of each TCGA patient was derived from a previously published study (2). A correlation analysis between gene expression and TMB or MSI was performed by using the “cor.test” command. All results were visualized by a radar chart, which was designed with the R-package “fmsb”.

### Drug Susceptibility Analysis

The NCI-60 database contains data on 60 different cancer cell lines from nine different types of tumors by using CellMiner interface (https://discover.nci.nih.gov/cellminer/). The NCI-60 cell line is currently the most widely used cancer cell sample group for anti-cancer drug testing. Drug sensitivity data and RNA-seq data were downloaded from the NCI-60 database to explore the relationship between genes and the sensitivity of common anti-tumor drugs.

### Gene Enrichment Analysis

Gene set variation analysis (GSVA) is a non-parametric and unsupervised method for evaluating the enrichment of transcriptome gene sets. GSVA was performed using the GSVA R package, to comprehensively score several gene sets of interest with a Poison distribution, and then converts gene-level changes into pathway-level changes. The gene set was downloaded from Molecular signatures database (v7.0 version), and the GSVA algorithm was used to comprehensively score each gene set to evaluate the potential biological function changes of different samples.

Gene Set Enrichment Analysis (GSEA) was used to identify the level of depletion or enrichment by using a predefined gene set and ranking the genes according to the degree of differential expression between two compared groups. In this study, GSEA analysis was performed through the “clusterprofiler” and “enrichplot” packages, and the possible molecular mechanisms of the prognostic differences were explored by comparing the differences in signal pathways between the high and low gene expression groups.

### WGCNA Analysis

The weighted correlation network analysis (WGCNA) was used to construct weighted gene co-expression network, and to explore the relationship between the gene network and biological traits, as well as the hub genes in the network. The “WGCNA” R package was used to construct a co-expression network of all genes, and the genes with the top 5000 variances were screened by this algorithm for further analysis. The soft thresholding power was set as 4. Then the adjacency matrix was transformed into a topological overlap matrix (TOM) to estimate the network connectivity, and the TOM-based dissimilarity matrix for hierarchical clustering. Subsequently, a hierarchical clustering tree was constructed by the correlation coefficient between genes, and different branches of the clustering tree represented different gene modules, and different colors represented different modules. Based on the weighted correlation coefficient, the genes were classified different modules based on the expression patterns, and genes with similar expression patterns were grouped into one module. In this way, tens of thousands of genes were divided into multiple modules by gene expression patterns.

### Predictive Model Construction

In this study, multivariate Cox regression analysis were performed to determine independent prognostic factors to construct the nomogram using the R software package. The calibration curve was plotted accordingly.

### Functional Verification of WASF2

In the study, siRNA targeting WASF2 was used to knockdown WASF2 mRNA expression. F-actin was visualized with phalloidin staining according to manufacturer’s instructions. CCK8 assay was performed to detect cell proliferation. Transwell and wound healing/scratch assays were used to evaluate cell invasion and migration. WASF2 expression in ovarian cancer tissues was evaluated by immunohistochemical staining. The detailed materials and methods were described in **Supplementary Materials and Methods**.

### Statistic

All statistical analyses were conducted using R language (version 4.1.1). The hazard ratios (HR) and 95% confidence intervals were calculated using univariate survival analysis. A Kaplan-Meier survival analysis was performed to estimate survival curve. The difference in WASF2 expression between different tumor stages was compared using the Kruskal-Wallis test. Pearson test was used for correlation analysis. The correlation between WASF2 expression and clinicalpathological characters was evaluated by Chi-square test (χ2). For normally distributed data, Student’s t-test was applied, whereas a Mann-Whitney U test was used for nonnormally distributed data. A two-sided *P*-value < 0.05 indicated a statistically significant difference.

## Results

### Pan-Cancer Expression Landscape of WASF2

**Figure 1:**
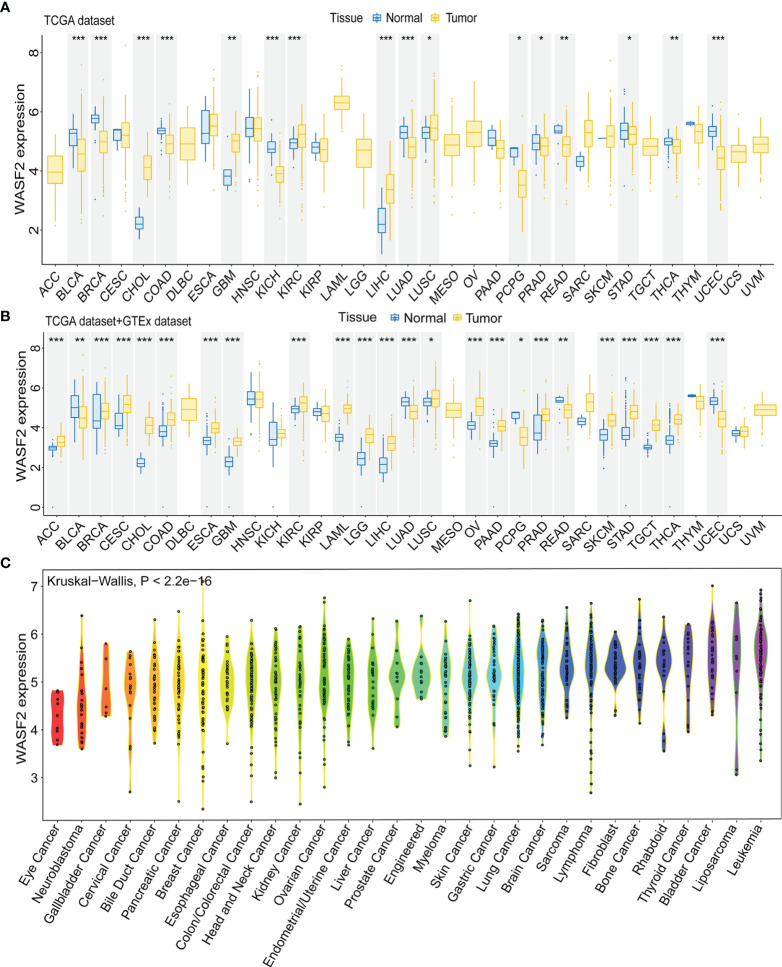
The WASF2 expression level in human pan-cancer analyses. **(A)** The mRNA level of WASF2 in TCGA. The color refers to the tumor (yellow) or normal (blue), respectively. **(B)** The WASF2 expression level in 33 types from the GTEx database and TCGA database. **(C)** WASF2 expression in 30 tumor cells from CCLE database. *P < 0.05; **P < 0.01; ***P < 0.001.

**Figure 2:**
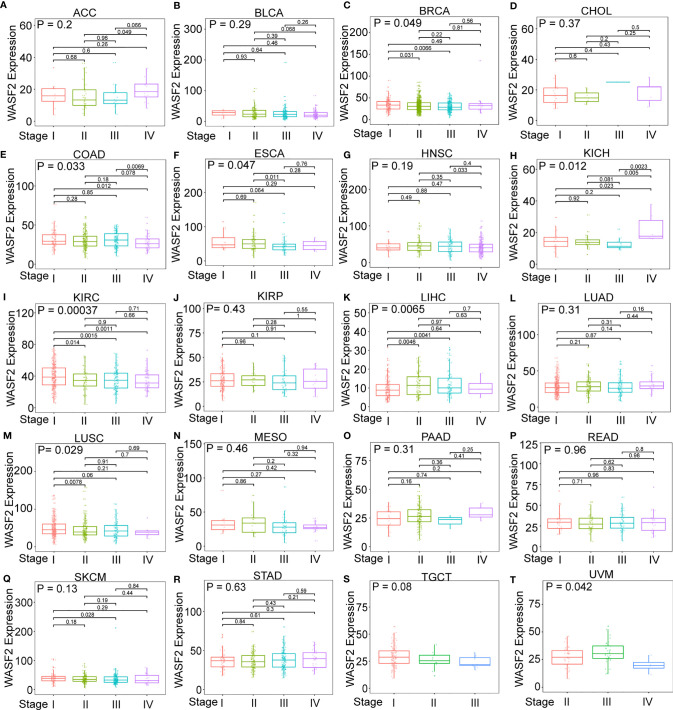
The box plot shows the association of WASF2 expression with pathological stages in **(A)** adrenocortical carcinoma (ACC), **(B)** bladder urothelial carcinoma (BLCA), **(C)** breast invasive carcinoma (BRCA), **(D)** cholangiocarcinoma (CHOL), **(E)** colon adenocarcinoma (COAD), **(F)** esophageal carcinoma (ESCA), **(G)** head and neck squamous cell carcinoma (HNSC), **(H)** kidney chromophobe (KICH), **(I)** kidney renal clear cell carcinoma (KIRC), **(J)** kidney renal papillary cell carcinoma (KIRP), **(K)** liver hepatocellular carcinoma (LIHC), **(L)** lung adenocarcinoma (LUAD), **(M)** lung squamous cell carcinoma (LUSC), **(N)** mesothelioma (MESO), **(O)** pancreatic adenocarcinoma (PAAD), **(P)** rectum adenocarcinoma (READ), **(Q)** skin cutaneous melanoma (SKCM), **(R)** stomach adenocarcinoma (STAD), **(S)** testicular germ cell tumors (TGCT), and **(T)** uveal melanoma (UVM). Kruskal-Wallis test was used to assess the significance of differences between groups, followed by pair wise comparisons using Dunn’s multiple comparisons test used to evaluate differences among groups.

First, the expression of WASF2 in 33 human cancers was analyzed by using TCGA and GTEx datasets. As shown in **Figure 1A**, WASF2 was generally highly expressed in 5 tumors, including CHOL, GBM, KIRC, LIHC, and LUSC. However, it was significantly lower in BLCA, BRCA, COAD, KICH, LUAD, PCPG, PRAD, READ, STAD, THCA, and UCEC compared with normal tissues. Due to the small number of normal tissue samples in TCGA database, the normal tissue data from the GTEx database and the tumor tissue data from TCGA database were combined to analyze the differences of WASF2 expression in 33 cancers. The results showed that WASF2 was abnormally expressed in 24 of these tumors. Specifically, WASF2 expression was higher in 19 cancer (ACC, BRCA, CESC, CHOL, COAD, ESCA, GBM, KIRC, LAML, LGG, LIHC, LUSC, OV, PAAD, PRAD, SKCM, STAD, TGCT, and THCA) and lower in 5 cancers (BLCA, LUAD, PCPG, READ, and UCEC) compared with the normal tissues (**Figure 1B** and **Supplement Figure 1A**). Furthermore, the expression levels of WASF2 in 30 tissue cell lines were analyzed using the data of tumor cell lines downloaded from the CCLE database. Results showed that WASF2 was expressed in all 30 kinds of tumor cell lines (**Figure 1C** and **Supplement Figure 1C**). In sum, these results suggested that WASF2 was abnormally expressed in different cancers. We also further found that WASF2 was related to the stage of various tumors, including BRCA, COAD, ESCA, KICH, KIRC, LIHC, LUSC and UVM (**Figure 2** and **Supplement Figure 1B**).

### Prognostic Value of WASF2 Across Cancers

**Figure 3:**
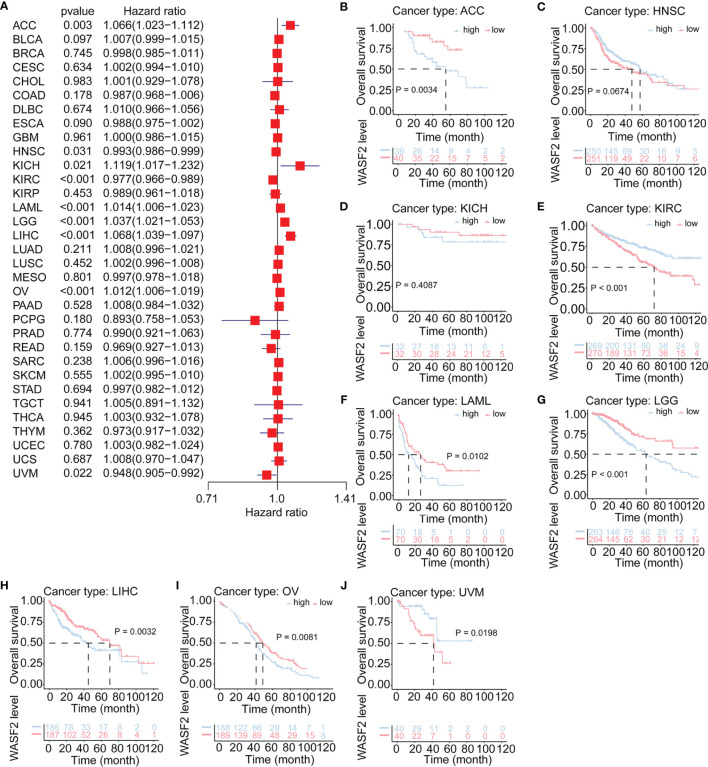
Association of WASF2 expression with patient overall survival (OS) in pan- cancer. **(A)** Forest plot of HR for the relationship between WASF2 expression and patient OS. **(B–J)** Kaplan-Meier analyses show the association between WASF2 expression and OS. Statistical significance was assessed using the log-rank test.

The relationship between WASF2 expression and the prognosis of patients in pan-cancer was analyzed. In the OS analysis, Cox regression identified that high WASF2 expression was a risk factor for ACC (*P* = 0.003, HR = 1.066), KICH (*P* = 0.021, HR = 1.119), LAML (*P* < 0.001, HR = 1.014), LGG (*P* < 0.001, HR = 1.037), LIHC (*P* < 0.001, HR = 1.068), and OV (*P* < 0.001, HR = 1.012); however, it appeared to be a protective factor in HNSC (*P* = 0.031, HR = 0.993), KIRC (*P* < 0.001, HR = 0.977), and UVM (*P* = 0.022, HR = 0.948), as shown in **Figure 3A**. KM survival curve showed that patients with higher WASF2 levels had a shorter OS compared with patients with lower WASF2 levels in ACC (*P* = 0.003), LAML (*P* < 0.010), LGG (*P* < 0.001), LIHC (*P* = 0.003) and OV (*P* = 0.008), whereas those with elevated WASF2 levels tended to be better OS to those with decreased WASF2 levels in KIRC (*P* < 0.001) and UVM (*P* = 0.020), as displayed in **Figures 3B**–**J**.

**Figure 4:**
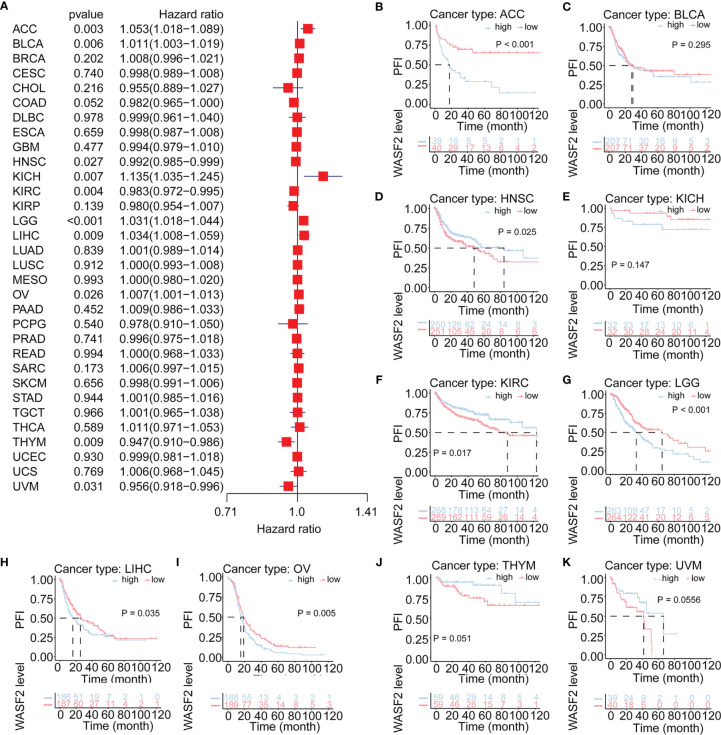
Association of WASF2 expression with patient progression-free interval (PFI) in pan- cancer. **(A)** Forest plot of HR for the relationship between WASF2 expression and patient PFI. **(B–K)** Kaplan-Meier analyses show the association between WASF2 expression and PFI. Statistical significance was assessed using the log-rank test.

Cox regression analysis of PFI identified high WASF2 expression was a risk factor for ACC (*P* = 0.003, HR = 1.053), BLCA (*P* = 0.006, HR = 1.011), KICH (*P* = 0.007, HR = 1.135), LGG (*P* < 0.001, HR = 1.031), LIHC (*P* = 0.009, HR = 1.034), and OV (*P* = 0.026, HR = 1.007); however, it was a protective factor in HNSC (*P* = 0.027, HR = 0.992), KIRC (*P* = 0.004, HR = 0.983), THYM (*P* = 0.009, HR = 0.947), and UVM (*P* = 0.031, HR = 0.956) (**Figure 4A**). The KM results demonstrated that patients with higher WASF2 expression had a poorer PFI compared to patients with lower WASF2 levels in ACC (*P* < 0.001), BLCA (*P* = 0.295), KICH (*P* = 0.147), LGG (*P* < 0.001), LIHC (*P* = 0.035) and OV (*P* = 0.005), while patients with increased WASF2 levels showed a superior PFI than those with decreased WASF2 levels in HNSC (*P* = 0.025), KIRC (*P* = 0.017), THYM (*P* = 0.051) and UVM (*P* = 0.055), as shown in **Figures 4B**–**K**.

### The Relationship Between WASF2 Expression and Immune Infiltration

**Figure 5:**
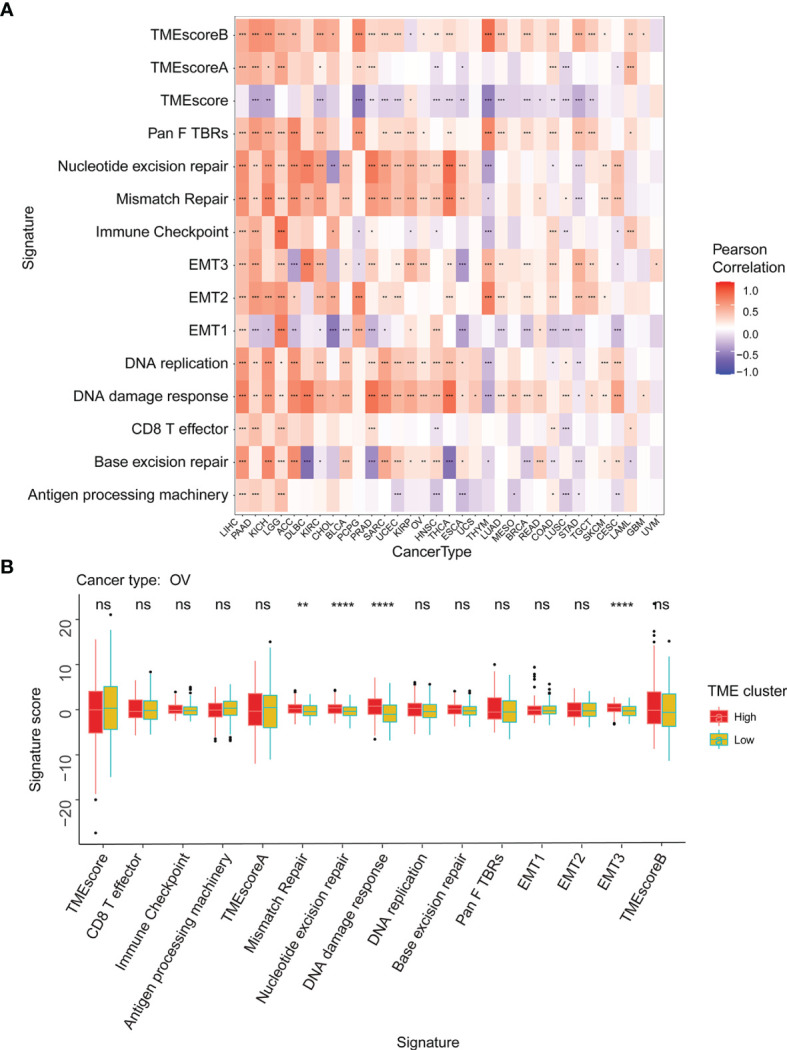
WASF2 expression is correlated with the TME. **(A)** Correlation between the expression of WASF2 and 15 TME processes. Red denotes a correlation coefficient > 0, whereas blue denotes a correlation coefficient < 0. **(B)** The statistical chart after using the CIBERSORT method shows the proportion difference of TME between WASF2 high and low expression groups in ovarian cancer. Red represents the high WASF2 expression group, yellow represents the low WASF2 expression group. *P < 0.05; **P < 0.01; ***P < 0.001. ns, no significant.

The tumor microenvironment (TME) is a complex non-tumor cell environment composed mainly of tumor-related fibroblasts, immune cells, extracellular matrix, a variety of growth factors, inflammatory factors, special physical and chemical characteristics, and cancer cells themselves (22). In recent years, the importance TME in the occurrence and development of cancer has attracted more and more attention. Therefore, it is important to further explore the pan-cancer relationship between WASF2 expression and TME. Our results showed that the expression of WASF2 was closely related to TME (**Figure 5A**). We conducted further TME analysis on OV, and the results showed that mismatch repair, EMT3, nucleotide excision repair, DNA damage response and other scores were significantly related to OV (**Figure 5B**).

**Figure 6:**
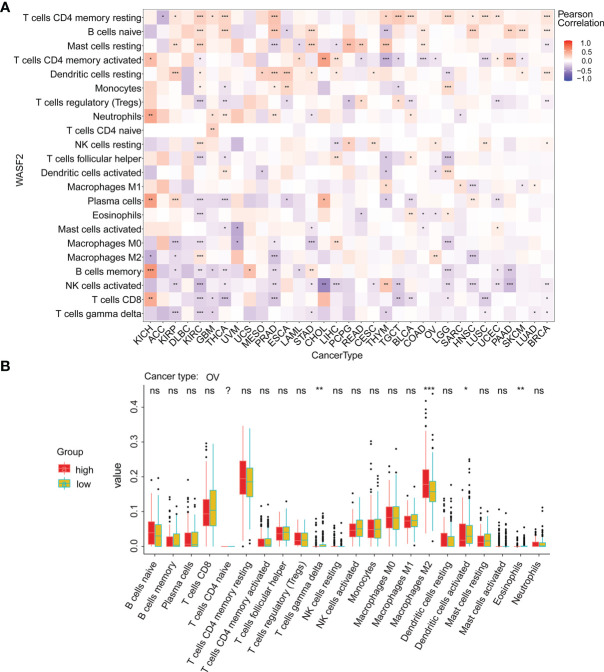
WASF2 expression is correlated with cancer immunity. **(A)** Correlation between the expression of WASF2 and infiltration by 22 types of immune cells in pan-cancer analysis. Red denotes a correlation coefficient > 0, whereas blue denotes a correlation coefficient < 0. **(B)** The statistical chart after using the CIBERSORT method shows the proportion difference of immune cell between WASF2 high and low expression groups in ovarian cancer. Red represents the high WASF2 expression group, yellow represents the low WASF2 expression group. *P < 0.05; **P < 0.01; ***P < 0.001. ns, no significant.

Our results also showed that the expression of WASF2 was closely related to immune infiltration. Among them, 14 cancers were significantly related to T cells CD4 memory resting, 11 cancers were significantly related to B cells naive, and 11 cancers were significantly related to mast cells resting (**Figure 6A**). We conducted further immune infiltration analysis on OV, and the results showed that T cells gamma delta, macrophages M2, dendritic cells activated, and eosinophils were significantly related to OV (**Figure 6B** and **Supplement Figure 2**). Next, the correlation between WASF2 level and infiltration of immune cells in pan-cancer was explored using Tumor Immune Estimation Resource (TIMER). The results were presented as a heat map. Red represent positive correlation and blue negative correlation (**Supplement Figures 3**, **4**).

### The Relationship Between WASF2 Expression and Key Regulatory Genes

**Figure 7:**
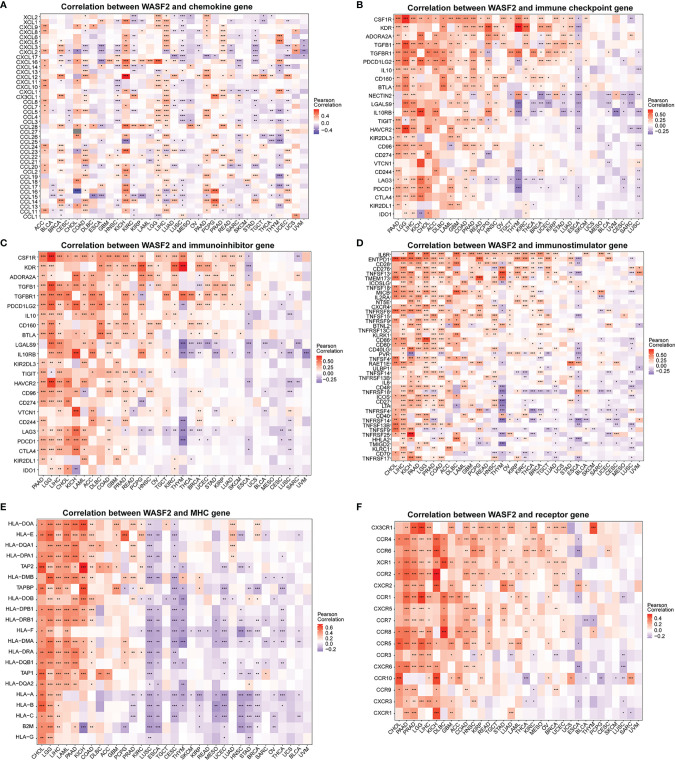
WASF2 expression is correlated with immune-related genes. **(A)** The correlation between WASF2 and chemokine gene. **(B)** The correlation between WASF2 and immune checkpoint gene. **(C)** The correlation between WASF2 and immunoinhibitor gene. **(D)** The correlation between WASF2 and immunostimulator gene. **(E)** The correlation between WASF2 and MHC gene. **(F)** The correlation between WASF2 and receptor gene. *P < 0.05; **P < 0.01; ***P < 0.001.

We further conducted correlation analysis to explore the relationship between WASF2 expression and 33 tumor immune-related genes. The genes analyzed included MHC, immune activating factors, immunosuppressive factors, chemokines, and chemokine receptor proteins. The results showed that almost all immune-related genes were significantly related to WASF2 (**Figure 7**). In addition, WASF2 has significant correlation with common tumor-related regulatory genes such as autophagy, DNA repair, ferroptosis, hypoxia, pyroptosis, and TGF-β signaling gene (**Supplement Figure 5**).

### The Relationship Between WASF2 Expression and TMB and MSI and With Drug Sensitivity

**Figure 8:**
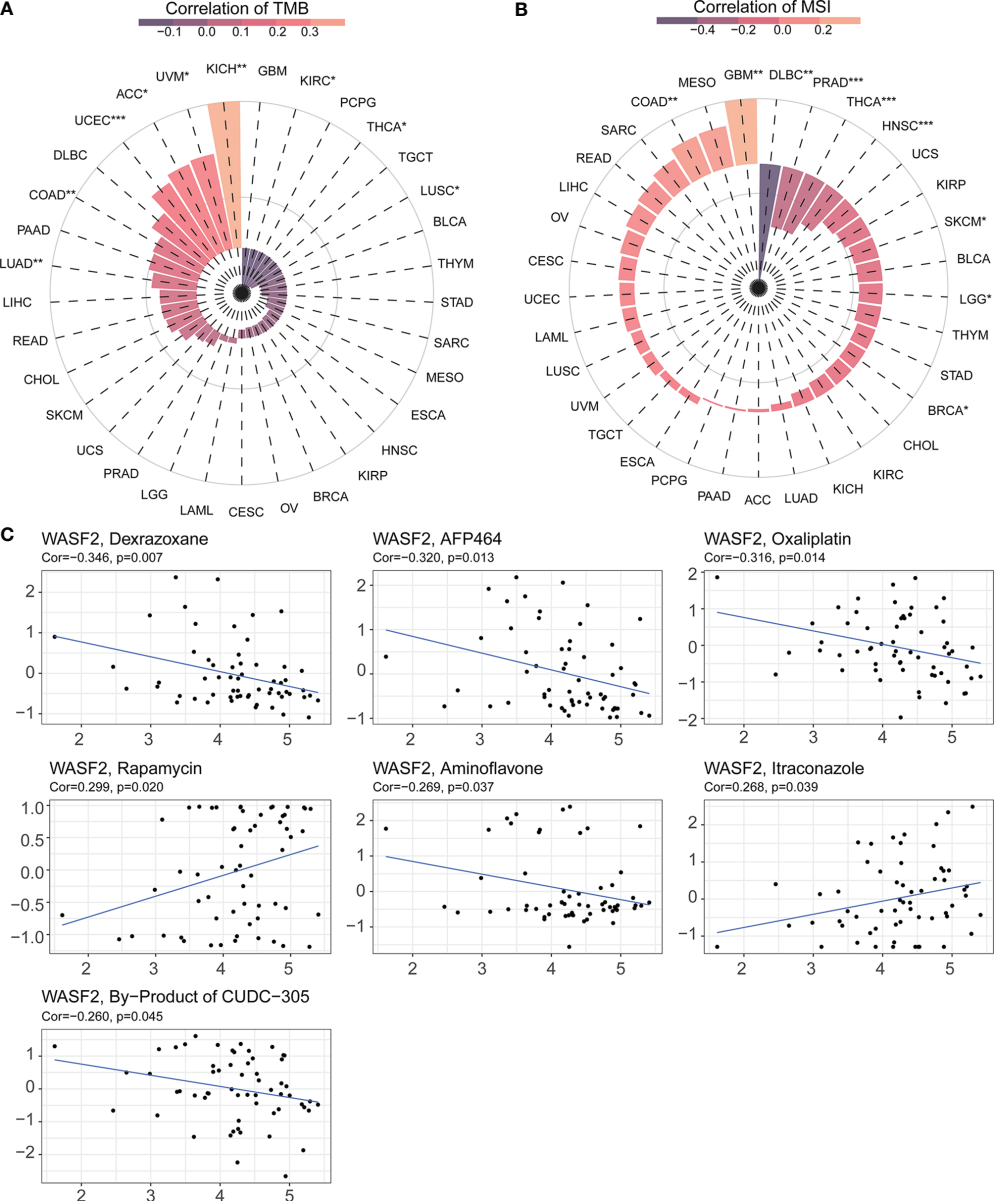
WASF2 expression is correlated with TMB, MSI and drug sensitivity. **(A)** Correlation analysis between WASF2 expression in pan-cancer and TMB described using Spearman’s rank correlation coefficient. **(B)** Correlation analysis between WASF2 expression in pan-cancer and MSI described using Spearman’s rank correlation coefficient. **(C)** Analysis of drug sensitivity associated with WASF2. The positive correlation means that the gene’s high expression is resistant to the drug, while the negative is the opposite. *P < 0.05; **P < 0.01; ***P < 0.001.

TMB and MSI were two emerging biomarkers related to immunotherapy response. We explored the relationship between WASF2 expression and TMB. The results showed that the expression level of WASF2 was significantly correlated with TMB tumors, including UCEC, COAD, LUSC, LUAD, ACC, KICH, KIRC, UVM, THCA, and there were significant differences (**Figure 8A**). In MSI, the WASF2 had significant differences in SKCM, PRAD, LGG, HNSC, COAD, BRCA, THCA, GBM, and DLBC (**Figure 8B**).

Early-stage tumors were treated with surgery combined with chemotherapy. The Cellminer database was used to explore the sensitivity of the WASF2 and common anti-tumor drugs, and we further calculated the correlation between the gene expression and the drug IC50. The results showed that high gene expression was correlated to resistance to the anti-tumor drug tolerance. Among them, the WASF2 was positively correlated with Rapamycin and Itraconazole, and negatively correlated with Dexrazoxane, AFP464, Oxaliplatin, Aminoflavone, By-Product of CUDC-305 (**Figure 8C**).

### Association of WASF2 Expression With GSVA and GSEA in Ovarian Cancer

**Figure 9:**
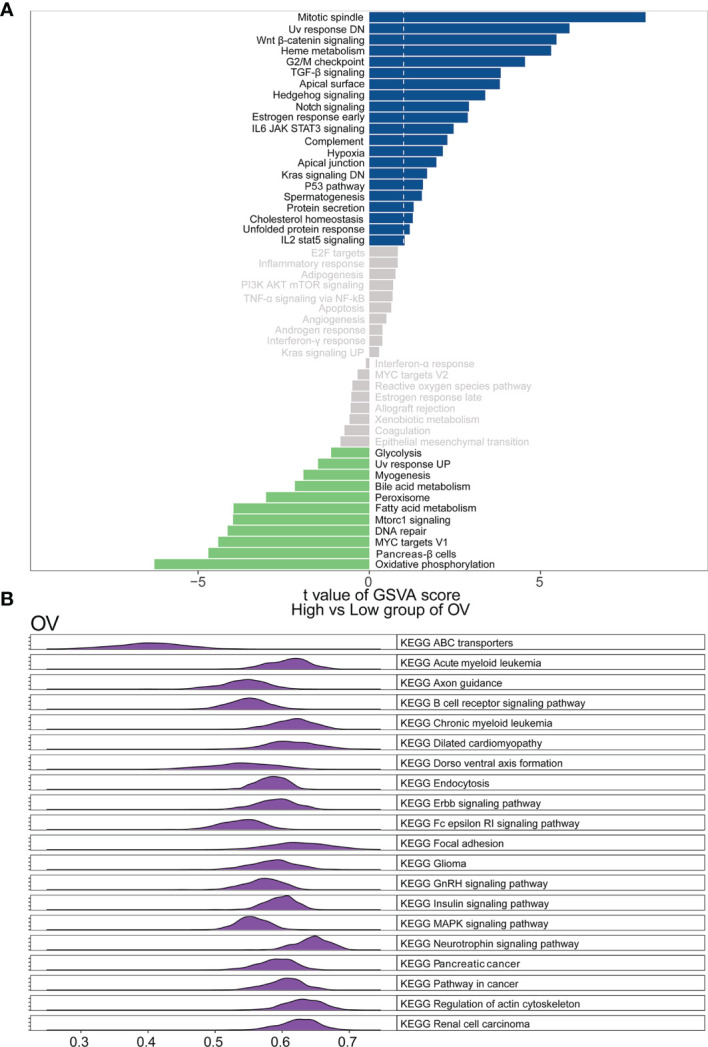
Function and pathway enrichment analysis of ovarian cancer. **(A)** Correlation analysis results of GSVA and WASF2 in ovarian cancer. **(B)** KEGG results of WASF2 GSEA in ovarian cancer.

To elucidate the detailed mechanism of the WASF2, further in-depth studies were required. Therefore, we first scored all tumors with GSVA analysis, and then divided the samples into two groups with high and low expression in each tumor separately using the median of the gene expression. The results showed that the high expression of WASF2 mainly focused on mitotic spindle, UV response DN, Wnt/β-catenin signaling, heme metabolism, and G2/M checkpoint in ovarian cancer (**Figure 9A**). The GSEA analysis of WASF2 in ovarian cancer was shown in the **Figure 9B** and **Supplement Figure 6**.

### Association of WASF2 Expression With WGCNA in Ovarian Cancer

**Figure 10:**
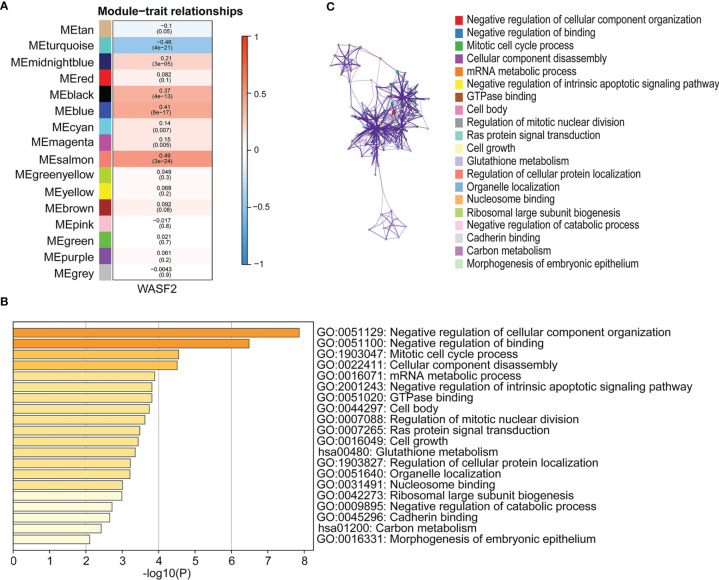
Association of WASF2 expression with WGCNA in ovarian cancer. **(A)** Module trait relationship (p-value) for detected modules (y-axis) in relation with traits (x-axis) for ovarian cancer. The relationships were colored based on the correlation between the identified module and traits. The color scale on the right demonstrate module-trait relationship from −1 (blue) to one (red), where blue represents strong negative correlation and red represents a strong positive correlation. **(B)** Functional annotations of MEsalmon module. Gene ontology (GO) and corresponding *P*-values are shown. **(C)** PPI network of GO enrichment analysis results.

WGCNA is a systematic biological approach to construct a scale-free network using gene expression data (23). So, we further performed WGCNA analysis based on the expression profile data of ovarian cancer, and constructed the co-expression network related to WASF2 in ovarian cancer. The soft power of β = 4 was selected as the appropriate soft‐thresholding for performing subsequent analyses by the function “sft$powerEstimate”. The gene modules were detected based on the TOM, and a total of 16 modules were finally detected. We further analyzed the module trait relationship and found that the MEsalmon module had the highest correlation (cor = 0.49, *P* = 3e^−24^) (**Figure 10A**). The Metascape website was used to perform enrichment analysis on the salmon module gene. The results revealed that these genes were mainly enriched in negative regulation of cellular component organization, negative regulation of binding, mitotic cell cycle process, cellular component disassembly, and mRNA metabolic process (**Figures 10B, C**).

### Clinical Application of a Nomogram Incorporating the WASF2 Gene

**Figure 11:**
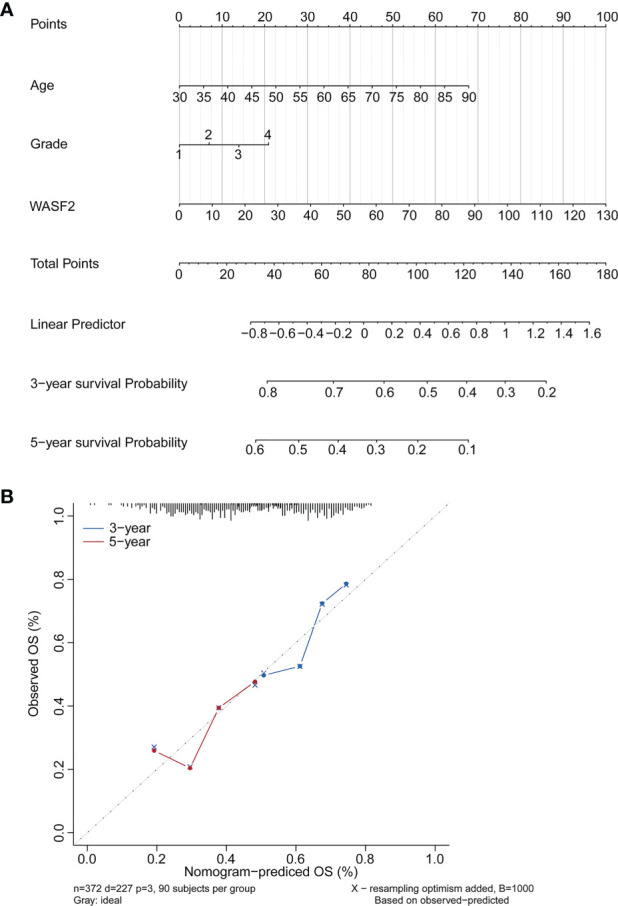
Establishment and validation of the prognostic nomogram. **(A)** Nomogram based on the WASF2 signature and clinical information for prediction of the 3- and 5-year OS in patients with ovarian cancer in the TCGA dataset. **(B)** The calibration curves is used to verify the consistency of predicted and actual 3-, 5-year outcomes.

Based on the findings of the Cox regression analyses, we further constructed a nomogram integrating the age, grade, and the expression of WASF2, to provide a quantitative method for clinicians to predict the probability of 3‐ and 5‐year OS in ovarian cancer patients (**Figure 11A**). To calculate the score, each prognostic parameter was projected upward to the value of the small ruler (points), with a higher number of total points indicating a worse outcome for the patient. In addition, the calibration curve for the 3‐ and 5‐year OS was plotted at the same time, and the nomogram had a good performance (**Figure 11B**).

### WASF2 Knockdown Suppresses the Proliferation, Migration and Invasion of Ovarian Cancer Cells

**Figure 12:**
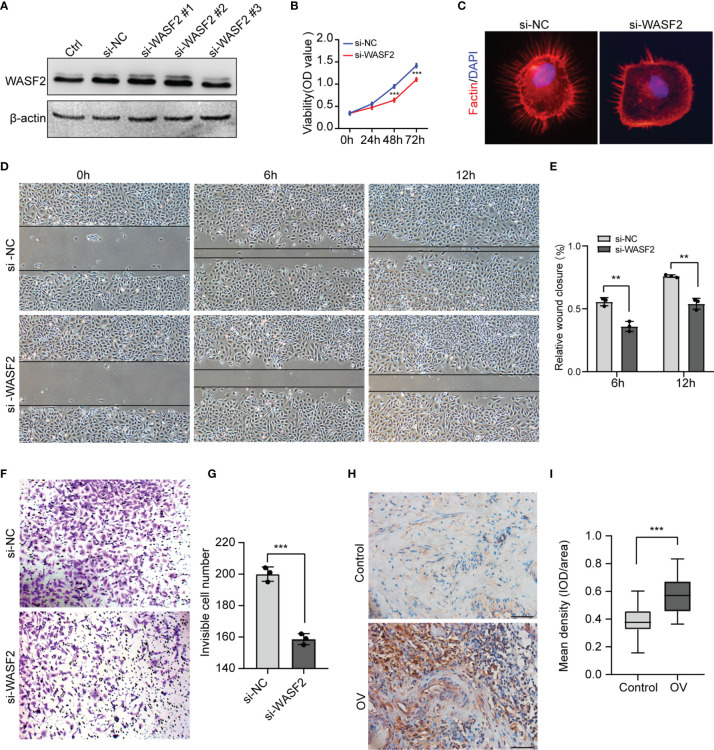
Knockdown of WASF2 inhibits the proliferation, migration and invasion capacities of ovarian cancer cells *in vitro*. **(A)** Western blot analysis of transfection efficiency of the siRNA. **(B)** Cell proliferation was detected by using the CCK8 proliferation reagent. **(C)** WASF2 knockdown suppressed filopodia formation. **(D, E)** Effect of WASF2 knockdown in cell migration was determined by wound healing assay and the percentage of scratch-width closure measured by quantifying the images the scratch assay at 0, 6 and 12 h after incubation. **(F, G)** Invasiveness of ovarian cancer cells analyzed by transwell invasion assay (magnification, ×200) and bar graph showing quantitative results of the transwell assay. **(H, I)** Representative immunohistochemical staining of ovarian cancer tissues using anti-WASF2 antibody showing high expression of WASF2. *P < 0.05; **P < 0.01; ***P < 0.001. Scale bars = 50μm in H.

To investigate the role of WASF2 in the migration and invasion of ovarian cancer cells, WASF2 expression was silenced using specific siRNAs (**Figure 12A**). The influence of silenced WASF2 on the proliferation of ovarian cancer cells was detected by the CCK8 assay, which showed that WASF2 knockdown significantly suppressed the proliferation of cells (**Figure 12B**). Meanwhile, we found that WASF2 knockdown inhibited the formation of filopodia (**Figure 12C**). Following WASF2 knockdown, cells exhibited a significantly slower closure of the wound area than control cells (**Figures 12D, E**) and the invasion potentials were significantly reduced compared with their respective control cells (**Figures 12F, G**). These results suggest that WASF2 serves an important role in the migration and invasion of ovarian cancer cells. Subsequent immunohistochemistry further confirmed WASF2 expression was significantly increased in ovarian cancer tissues (**Figures 12H, I**).

## Discussion

In this study, we conducted a comprehensive methodology for pan-cancer analysis and investigated the roles of the WASF2 in cancers. Our results indicated that WASF2 was highly expressed in a variety of cancers, and its expression level was closely related to many tumor progression, stage and prognosis. Due to the significance of the TME in malignancies, the relationship between WASF2 and the infiltration of immune cells was worth further analysis. We found that WASF2 expression correlated with cancer immunity, immune regulatory genes, TMB and MSI. Then, we performed GSVA, GSEA and WGCNA analysis to further elucidate the potential mechanisms in ovarian cancer. In addition, we constructed a nomogram that could be used for assessing the survival probability of patients with ovarian cancer based on age, stage and the expression of WASF2. Finally, our *in vitro* experiments demonstrated that silencing WASF2 inhibited the proliferation, migration, and invasiveness of ovarian cancer cells, and confirmed that WASF2 were highly expressed in ovarian cancer by immunohistochemistry.

Our current study showed that the WASF2 was highly expressed in 19 cancers and low in 5 cancers. The results for BRCA (13), COAD (24), GBM (25), LGG (26) and STAD (27) were similar to the previous research. However, previous studies have reported that WASF2 expression was elevated in LUAD (12), which was inconsistent with our current results and required more experimental verification. We found that the expression of WASF2 was significantly increased in ovarian cancer, suggesting that WASF2 might play an important role in the development of ovarian cancer, which would provide new insights for anti-tumor therapy. As is known, critical analysis of factors involved in survival and prognosis can facilitate treatment decisions for patients and clinicians (28). Therefore, we also analyzed the relationship between WASF2 expression and survival. We found that high expression of WASF2 was accompanied by poor prognosis and short survival time for ACC, KICH, LAML, LGG, LIHC, and OV. However, our study identified that the high expression of WASF2 appeared to be a protective factors for HNSC, KIRC, THYM, and UVM. Despite of this, the elucidation of the specific mechanism still required further investigation. In conclusion, pan-cancer analysis of WASF2 was valuable for identifying differential expressions and the roles of WASF2 in many cancer types.

Nowadays, the metabolism and microenvironment of tumors are receiving more and more attention (29). And TME has emerged as a critical component in understanding the tumorigenesis and tumor progression (30, 31). In addition, TME, especially the immune microenvironment composed of tumor-infiltrating lymphocytes (B cells and T cells) and other immune cells (macrophages, neutrophils, and dendritic cells), has been taken into consideration for the tumor therapy (32–36). Studies have shown that immune cells may have both tumor promoting and tumor inhibiting roles in immune regulation (37–41). On the one hand, under normal circumstances, immune cells play a crucial role as a component of the innate immune system killing cancer cells and fighting infections (42). On the other hand, cancer cells have been shown to utilize a variety of mechanisms to escape immune surveillance (43, 44). Past research indicates that macrophages in TME can polarize to M2 macrophages, and M2 macrophages can further shape immune microenvironment that promotes tumor progression (45). Besides, the CD3^+^, CD4^+^, and CD8^+^ cell subsets of T lymphocytes, as well as the CD4^+^/CD8^+^ ratio, play a significant role in antitumor immunity (3). Neutrophils can help tumor cells escape immune surveillance by mediating tumor angiogenesis as well as promoting cancer cell invasion, proliferation, and metastasis (46, 47). However, there are few studies on the relationship between WASF2 and immune cell infiltration. In our analysis, we discovered that 14 malignancies were strongly associated with CD4^+^ T cells, 11 cancers were significantly associated with B cells, and 11 cancers were significantly associated with resting mast cells. In addition, our analysis showed that WASF2 expression levels in ovarian cancer were correlated with four different types of immune-infiltrating cells (T cells gamma delta, Macrophages M2, Dendritic cells activated, Eosinophils). At present, there is still no systematic molecular mechanism for the relationship between WASF2 and immune cells in ovarian cancer, which is innovative and worthy of further exploration. Our research further clarified that WASF2 had a wider range of tumor applicability, and confirmed that WASF2 expression was closely related to the biological processes of immune cells and immune-related molecules in most cancers. Besides that, our research also revealed the co-expression of WASF2 with genes encoding chemokine, immune checkpoint, immunosuppression, immune activation, MHC, and receptor proteins. These results indicated that WASF2 may serve an important role in the TME, affecting the prognosis of patients, and may be involved in the development of cancers, especially in ovarian cancer.

Recently, an increasing number of studies have demonstrated that TMB and immune cell infiltration played a vital role in immunotherapy response across multiple cancer types (48, 49). Previous studies showed that blood TMB can be used as a predictive biomarker in patients with non-small cell lung cancer and platinum-resistant recurrent ovarian cancer (50, 51). In addition, some studies have found there was a significant negative correlation between TMB values and prognosis prediction performances (52). For example, patients with low TMB had a worse outcome than those with high TMB (53). MSI has emerged as a potential biomarker for predicting therapeutic response to immune checkpoint inhibitors (ICI) (54). Only the MSI fraction of colorectal cancers has showed a good response to ICI treatment (55). In our study, we found that the expression of WASF2 was significantly associated with TMB in 9 cancer types and MSI in 9 cancer types. This might indicate that the aberrant WASF2 expression wound affect the TMB and MSI of cancer, thus impacting the patient response to ICI. Those provided a useful reference in the immunotherapy of cancer.

CellMiner is a database of genomic and pharmacologic tools for identifying drug patterns and transcripts in the NCI-60 cell line (56). We found that the high expression of WASF2 was positively correlated with the sensitivity of a few drugs approved by the FDA (Rapamycin and Itraconazole), and negatively correlated with 5 drugs (Dexrazoxane, AFP464, Oxaliplatin, Aminoflavone, and By-Product of CUDC-305). Our data showed that the expression of WASF2 in cancer cells could be regarded as a biological indicator to predict the sensitivity or resistance of cells to drugs, and provide effective support for subsequent basic research or clinical applications.

Next, we performed GSVA and GSEA analysis to explore underlying biological mechanisms in ovarian cancer. Our enrichment analysis indicated that WASF2 could potentially impact etiology or pathogenesis of ovarian cancer by functioning in mitotic spindle, genes down-regulated in response to ultraviolet radiation, Wnt/β-catenin signaling, heme metabolism, and G2/M checkpoint. The WGCNA results revealed that these genes were mainly enriched in negative regulation of cellular component organization, negative regulation of binding, mitotic cell cycle process, cellular component disassembly, and mRNA metabolic process. These results also suggested that WASF2 participated in various biological processes that promoted cancers development. This is in line with results from previous some experimental studies (9, 27, 57). Cohen et al. found that WASF2 complex regulates filamentous actin content and a decrease in actin levels is sufficient to elevate Wnt/β-catenin signaling (58). Numerous studies showed that the overexpression of WASF2 significantly promoted migration and invasion tumor cells and that tumor cell migration and invasion can be inhibited by downregulating WASF2 (14, 59–61).

Accordingly, our functional studies demonstrated that downregulating WASF2 could suppress the proliferation, migration and invasion of ovarian cancer cells. There were still some limitations in this study; for example, the specific molecular mechanism was not deeply studied. In addition, the precise regulatory mechanisms of WASF2 in the tumor microenvironment require further exploration in future studies. Finally, clinical samples were relatively small, and we will expand the sample size for analysis. And external validation was needed to conduct to verify the accuracy and reliability of prediction models in the future.

In conclusion, our comprehensive pan-cancer analysis showed the characterization WASF2 within tissue and cell line. Moreover, the expression of WASF2 has been associated with the risk and prognosis of multiple cancers. Based on the results of the present study, we hypothesized that WASF2 expression was associated with immune infiltration, and a potential marker of the TME. In addition, WASF2 expression was associated with TMB, MSI, and antitumor drugs sensitivity across various cancer types. Functional bioinformatics analysis demonstrated that the WASF2 may be involved in several signaling pathways and biological processes of ovarian cancer. Finally, a risk factor model was found to be predictive for OS in ovarian cancer based on the expression of WASF2. Meanwhile, it was further confirmed by immunohistochemistry that the higher expression of WASF2 in patients diagnosed with ovarian cancer. In summary, these results of this study might help elucidate the role of WASF2 in tumor occurrence and development.

## Data Availability Statement

The original contributions presented in the study are included in the article/**Supplementary Material**. Further inquiries can be directed to the corresponding author.

## Ethics Statement

The studies involving human participants were reviewed and approved by the Medical Ethics Committee of the First Affiliated Hospital of Jinan University. The patients/participants provided their written informed consent to participate in this study.

## Author Contributions

XY, YD, and LS contributed equally to this article. All authors contributed to the article and approved the submitted version.

## Funding

This study was supported by the Pilot Specialist Construction Project-Obstetrics and Gynecology (grant no. 711008).

## Conflict of Interest

The authors declare that the research was conducted in the absence of any commercial or financial relationships that could be construed as a potential conflict of interest.

## Publisher’s Note

All claims expressed in this article are solely those of the authors and do not necessarily represent those of their affiliated organizations, or those of the publisher, the editors and the reviewers. Any product that may be evaluated in this article, or claim that may be made by its manufacturer, is not guaranteed or endorsed by the publisher.

## References

[1] Ferlay J Colombet M Soerjomataram I Mathers C Parkin DM Piñeros M. Estimating the Global Cancer Incidence and Mortality in 2018: GLOBOCAN Sources and Methods. Int J Cancer (2019) 144(8):1941–53. doi: 10.1002/ijc.31937 30350310

[2] Arya N Sardana V Saxena M Rangarajan A Katti DS. Recapitulating Tumour Microenvironment in Chitosan-Gelatin Three-Dimensional Scaffolds: An Improved In Vitro Tumour Model. J Royal Soc Interface (2012) 9(77):3288. doi: 10.1098/rsif.2012.0564 PMC3481596 22977099

[3] Farolfi A Gurioli G Fugazzola P Burgio SL Giorgi UD. Immune System and DNA Repair Defects in Ovarian Cancer: Implications for Locoregional Approaches. Int J Mol Sci (2019) 20(10):2569. doi: 10.3390/ijms20102569 PMC6566239 31130614

[4] Petrowsky H Fritsch R Guckenberger M De Oliveira ML Dutkowski P Clavien PA. Modern Therapeutic Approaches for the Treatment of Malignant Liver Tumours. Nat Rev Gastroenterol Hepatol (2020) 17(12):755–72. doi: 10.1038/s41575-020-0314-8 32681074

[5] Nam GH Choi Y Kim GB Kim S Kim SA Kim IS. Emerging Prospects of Exosomes for Cancer Treatment: From Conventional Therapy to Immunotherapy. Adv Mater (2020) 32(51):e2002440. doi: 10.1002/adma.202002440 33015883

[6] Derynck R Turley SJ Akhurst RJ. Tgfβ Biology in Cancer Progression and Immunotherapy. Nat Rev Clin Oncol (2021) 18(1):9–34. doi: 10.1038/s41571-020-0403-1 32710082 PMC9721352

[7] Gong N Sheppard NC Billingsley MM June CH Mitchell MJ. Nanomaterials for T-Cell Cancer Immunotherapy. Nat Nanotechnol (2021) 16(1):25–36. doi: 10.1038/s41565-020-00822-y 33437036

[8] Rottner K Stradal TE. Actin Dynamics and Turnover in Cell Motility. Curr Opin Cell Biol (2011) 23(5):569–78. doi: 10.1016/j.ceb.2011.07.003 21807492

[9] Tang Q Schaks M Koundinya N Yang C Pollard LW Svitkina TM. WAVE1 and WAVE2 Have Distinct and Overlapping Roles in Controlling Actin Assembly at the Leading Edge. Mol Biol Cell (2020) 31(20):2168–78. doi: 10.1091/mbc.E19-12-0705 PMC7550694 32697617

[10] Moulding DA Record J Malinova D Thrasher AJ. Actin Cytoskeletal Defects in Immunodeficiency. Immunol Rev (2013) 256(1):282–99. doi: 10.1111/imr.12114 PMC3884764 24117828

[11] Matalon O Reicher B Barda-Saad M. Wiskott-Aldrich Syndrome Protein–Dynamic Regulation of Actin Homeostasis: From Activation Through Function and Signal Termination in T Lymphocytes. Immunol Rev (2013) 256(1):10–29. doi: 10.1111/imr.12112 24117810

[12] Semba S Iwaya K Matsubayashi J Serizawa H Kataba H Hirano T. Coexpression of Actin-Related Protein 2 and Wiskott-Aldrich Syndrome Family Verproline-Homologous Protein 2 in Adenocarcinoma of the Lung. Clin Cancer Res (2006) 12(8):2449–54. doi: 10.1158/1078-0432.Ccr-05-2566 16638851

[13] Iwaya K Norio K Mukai K. Coexpression of Arp2 and WAVE2 Predicts Poor Outcome in Invasive Breast Carcinoma. Mod Pathol (2007) 20(3):339–43. doi: 10.1038/modpathol.3800741 17277766

[14] Taniuchi K Furihata M Naganuma S Saibara T. WAVE2 is Associated With Poor Prognosis in Pancreatic Cancers and Promotes Cell Motility and Invasiveness *via* Binding to ACTN4. Cancer Med (2018) 7(11):5733–51. doi: 10.1002/cam4.1837 PMC6246955 30353690

[15] Yang LY Tao YM Ou DP Wang W Chang ZG Wu F. Increased Expression of Wiskott-Aldrich Syndrome Protein Family Verprolin-Homologous Protein 2 Correlated With Poor Prognosis of Hepatocellular Carcinoma. Clin Cancer Res (2006) 12(19):5673–9. doi: 10.1158/1078-0432.Ccr-06-0022 17020969

[16] Kurisu S Suetsugu S Yamazaki D Yamaguchi H Takenawa T. Rac-WAVE2 Signaling is Involved in the Invasive and Metastatic Phenotypes of Murine Melanoma Cells. Oncogene (2005) 24(8):1309–19. doi: 10.1038/sj.onc.1208177 15608687

[17] DeBerardinis RJ. Tumor Microenvironment, Metabolism, and Immunotherapy. N Engl J Med (2020) 382(9):869–71. doi: 10.1056/NEJMcibr1914890 32101671

[18] Shihab I Khalil BA Elemam NM Hachim IY Maghazachi AA. Understanding the Role of Innate Immune Cells and Identifying Genes in Breast Cancer Microenvironment. Cancers (2020) 12(8):2226. doi: 10.3390/cancers12082226 PMC7464944 32784928

[19] Kritikou JS Dahlberg CI Baptista MA Wagner AK Banerjee PP Gwalani LA. IL-2 in the Tumor Microenvironment is Necessary for Wiskott-Aldrich Syndrome Protein Deficient NK Cells to Respond to Tumors *In Vivo* . Sci Rep (2016) 21(8):630636. doi: 10.1038/srep30636 PMC4967920 27477778

[20] Nolz JC Nacusi LP Segovis CM Medeiros RB Mitchell JS Shimizu Y. The WAVE2 Complex Regulates T Cell Receptor Signaling to Integrins *via* Abl- and CrkL-C3G-Mediated Activation of Rap1. J Cell Biol (2008) 182(6):1231–44. doi: 10.1083/jcb.200801121 PMC2542481 18809728

[21] Liu M Zhang J Pinder BD Liu Q Wang D Yao H. WAVE2 Suppresses mTOR Activation to Maintain T Cell Homeostasis and Prevent Autoimmunity. Science (2021) 371(6536):4544. doi: 10.1126/science.aaz4544 33766857

[22] Casey SC Amedei A Aquilano K Azmi AS Benencia F Bhakta D. Cancer Prevention and Therapy Through the Modulation of the Tumor Microenvironment. Semin Cancer Biol (2015) 35 Suppl(Suppl):S199–223. doi: 10.1016/j.semcancer.2015.02.007 25865775 PMC4930000

[23] Yin K Zhang Y Zhang S Bao Y Guo J Zhang G. Using WeightedGgene Co-Expression Network Analysis to Identify Key Modules and Hub Genes in Tongue Squamous Cell Carcinoma. Medicine (2019) 98(37):e17100. doi: 10.1097/MD.0000000000017 31517839 PMC6750333

[24] Iwaya K Oikawa K Semba S Tsuchiya B Mukai Y Otsubo T. Correlation Between Liver Metastasis of the Colocalization of Actin-Related Protein 2 and 3 Complex and WAVE2 in Colorectal Carcinoma. Cancer Sci (2007) 98(7):992–9. doi: 10.1111/j.1349-7006.2007.00488.x PMC11158612 17459058

[25] Shimamura S Sasaki K Tanaka M. The Src Substrate SKAP2 Regulates Actin Assembly by Interacting With WAVE2 and Cortactin Proteins. J Biol Chem (2013) 288(2):1171–83. doi: 10.1074/jbc.M112.386722 PMC3543001 23161539

[26] Zhou T Wang CH Yan H Zhang R Zhao JB Qian CF. Inhibition of the Rac1-WAVE2-Arp2/3 Signaling Pathway Promotes Radiosensitivity *via* Downregulation of Cofilin-1 in U251 Human Glioma Cells. Mol Med Rep (2016) 13(5):4414–20. doi: 10.3892/mmr.2016.5088 27052944

[27] Yao Q Cao Z Tu C Zhao Y Liu H Zhang S. MicroRNA-146a Acts as a Metastasis Suppressor in Gastric Cancer by Targeting WASF2. Cancer Lett (2013) 335(1):219–24. doi: 10.1016/j.canlet.2013.02.031 23435376

[28] Baker S Bakunina K Duijm M Hoogeman MS Cornelissen R Antonisse I. Development and External Validation of a Nomogram to Predict Overall Survival Following Stereotactic Body Radiotherapy for Early-Stage Lung Cancer. Radiat Oncol (2020) 15(1):89. doi: 10.1186/s13014-020-01537-z 32321553 PMC7178957

[29] Deng F Zhou R Lin C Yang S Wang H Li W. Tumor-Secreted Dickkopf2 Accelerates Aerobic Glycolysis and Promotes Angiogenesis in Colorectal Cancer. Theranostics (2019) 9(4):1001–14. doi: 10.7150/thno.30056 PMC6401398 30867812

[30] Wan G Xie W Liu Z Xu W Lao Y Huang N. Hypoxia-Induced MIR155 is a Potent Autophagy Inducer by Targeting Multiple Players in the MTOR Pathway. Autophagy (2014) 10(1):70–9. doi: 10.4161/auto.26534 PMC4389881 24262949

[31] Rhim AD Oberstein PE Thomas DH Mirek ET Palermo CF Sastra SA. Stromal Elements Act to Restrain, Rather Than Support, Pancreatic Ductal Adenocarcinoma. Cancer Cell (2014) 25(6):735–47. doi: 10.1016/j.ccr.2014.04.021 PMC4096698 24856585

[32] Lakins MA Ghorani E Munir H Martins CP Shields JD. Cancer-Associated Fibroblasts Induce Antigen-Specific Deletion of CD8 (+) T Cells to Protect Tumour Cells. Nat Commun (2018) 9(1):948. doi: 10.1038/s41467-018-03347-0 29507342 PMC5838096

[33] Lane RS Femel J Breazeale AP Loo CP Thibault G Kaempf A. Ifnγ-Activated Dermal Lymphatic Vessels Inhibit Cytotoxic T Cells in Melanoma and Inflamed Skin. J Exp Med (2018) 215(12):3057–74. doi: 10.1084/jem.20180654 PMC6279400 30381467

[34] Zakharia Y Bhattacharya A Rustum YM. Selenium Targets Resistance Biomarkers Enhancing Efficacy While Reducing Toxicity of Anti-Cancer Drugs: Preclinical and Clinical Development. Oncotarget (2018) 9(12):10765–83. doi: 10.18632/oncotarget.24297 PMC5828194 29535842

[35] Junttila MR de Sauvage FJ. Influence of Tumour Micro-Environment Heterogeneity on Therapeutic Response. Nature (2013) 501(7467):346–54. doi: 10.1038/nature12626 24048067

[36] Wang Z Song K Zhao W Zhao Z. Dendritic Cells in Tumor Microenvironment Promoted the Neuropathic Pain *via* Paracrine Inflammatory and Growth Factors. Bioengineered (2020) 11(1):661–78. doi: 10.1080/21655979.2020.1771068 PMC8291888 32434423

[37] Chen X Litzenburger UM Wei Y Schep AN LaGory EL Choudhry H. Joint Single-Cell DNA Accessibility and Protein Epitope Profiling Reveals Environmental Regulation of Epigenomic Heterogeneity. Nat Commun (2018) 9(1):4590. doi: 10.1038/s41467-018-07115-y 30389926 PMC6214962

[38] Chen F Zhang Y Bossé D Lalani AA Hakimi AA Hsieh JJ. Pan-Urologic Cancer Genomic Subtypes That Transcend Tissue of Origin. Nat Commun (2017) 8(1):199. doi: 10.1038/s41467-017-00289-x 28775315 PMC5543131

[39] Qian DC Xiao X Byun J Suriawinata AA Her SC Amos CI. PI3K/Akt/mTOR Signaling and Plasma Membrane Proteins Are Implicated in Responsiveness to Adjuvant Dendritic Cell Vaccination for Metastatic Colorectal Cancer. Clin Cancer Res (2017) 23(2):399–406. doi: 10.1158/1078-0432.Ccr-16-0623 27435399 PMC5611841

[40] Berntsson J Nodin B Eberhard J Micke P Jirström K. Prognostic Impact of Tumour-Infiltrating B Cells and Plasma Cells in Colorectal Cancer. Int J Cancer (2016) 139(5):1129–39. doi: 10.1002/ijc.30138 27074317

[41] Brunner M Maier K Rümmele P Jacobsen A Merkel S Benard A. Upregulation of CD20 Positive B-Cells and B-Cell Aggregates in the Tumor Infiltration Zone Is Associated With Better Survival of Patients With Pancreatic Ductal Adenocarcinoma. Int J Mol Sci (2020) 21(5):1779. doi: 10.3390/ijms21051779 PMC7084265 32150869

[42] Gonzalez H Hagerling C Werb Z. Roles of the Immune System in Cancer: From Tumor Initiation to Metastatic Progression. Genes Dev (2018) 32(19-20):1267–84. doi: 10.1101/gad.314617.118 PMC6169832 30275043

[43] Su W Han HH Wang Y Zhang B Giancotti FG. The Polycomb Repressor Complex 1 Drives Double-Negative Prostate Cancer Metastasis by Coordinating Stemness and Immune Suppression. Cancer Cell (2019) 36(2):139–55. doi: 10.1016/j.ccell.2019.06.009 PMC7210785 31327655

[44] Chen YL Lin HW Sun NY Yie JC Hung HC Chen CA. mTOR Inhibitors Can Enhance the Anti-Tumor Effects of DNA Vaccines Through Modulating Dendritic Cell Function in the Tumor Microenvironment. Cancers (Basel) (2019) 11(5):617. doi: 10.3390/cancers11050617 PMC6562783 31052575

[45] Ma J Zhang JK Yang D Ma XX. Identification of Novel Prognosis-Related Genes in the Endometrial Cancer Immune Microenvironment. Aging (Albany NY) (2020) 12(21):22152–73. doi: 10.18632/aging.104083 PMC7695382 33159014

[46] Zhao Y Huang X Ding TW Gong Z. Enhanced Angiogenesis, Hypoxia and Neutrophil Recruitment During Myc-Induced Liver Tumorigenesis in Zebrafish. Sci Rep (2016) 6(1):1–12. doi: 10.1038/srep31952 27549025 PMC4994033

[47] Hua X Long ZQ Zhang YL Wen W Guo L Xia W. Prognostic Value of Preoperative Systemic Immune-Inflammation Index in Breast Cancer: A Propensity Score-Matching Study. Front Oncol (2020) 9(1):10580. doi: 10.3389/fonc.2020.00580 PMC7186330 32373539

[48] Grosser R Cherkassky L Chintala N Adusumilli PS. Combination Immunotherapy With CAR T Cells and Checkpoint Blockade for the Treatment of Solid Tumors. Cancer Cell (2021) 36(5):471–82. doi: 10.1016/j.ccell.2019.09.006 PMC7171534 31715131

[49] Marta T Uhlenbrock F Claus C Herzig P Zippelius A. Fibroblast Activation Protein-Targeted-4-1BB Ligand Agonist Amplifies Effector Functions of Intratumoral T Cells in Human Cancer. J Immunother Cancer (2020) 8(2):e000238. doi: 10.1136/jitc-2019-000238 32616554 PMC7333869

[50] Hu-Lieskovan S Bhaumik S Dhodapkar K Grivel JCJB Maecker HT. SITC Cancer Immunotherapy Resource Document: A Compass in the Land of Biomarker Discovery. J Immunother Cancer (2020) 8(2):e000705. doi: 10.1136/jitc-2020-000705 33268350 PMC7713206

[51] Morse CB Elvin JA Gay LM Liao JB. Elevated Tumor Mutational Burden and Prolonged Clinical Response to Anti-PD-L1 Antibody in Platinum-Resistant Recurrent Ovarian Cancer. Gynecol Oncol Rep (2017) 21(3):2178–80. doi: 10.1016/j.gore.2017.06.013 PMC5510487 28736741

[52] Huang Z Johnson TS Han Z Helm B Cao S Zhang C. Deep Learning-Based Cancer Survival Prognosis From RNA-Seq Data: Approaches and Evaluations. BMC Med Genomics (2020) 13(Suppl 5):41. doi: 10.1186/s12920-020-0686-1 32241264 PMC7118823

[53] Lv J Zhu Y Ji A Zhang Q Liao G. Mining TCGA Database for Tumor Mutation Burden and Their Clinical Significance in Bladder Cancer. Biosci Rep (2020) 40(4):BSR20194337. doi: 10.1042/bsr20194337 32239176 PMC7178217

[54] Zheng K Wan H Zhang J Shan G Chai N Li D. A Novel NGS-Based Microsatellite Instability (MSI) Status Classifier With 9 Loci for Colorectal Cancer Patients. J Transl Med (2020) 18(1):215. doi: 10.1186/s12967-020-02373-1 32466784 PMC7257555

[55] Ganesh K Stadler ZK Cercek A Mendelsohn RB Shia J Segal NH. Immunotherapy in Colorectal Cancer: Rationale, Challenges and Potential. Nat Rev Gastroenterol Hepatol (2019) 16(6):361–75. doi: 10.1038/s41575-019-0126-x PMC7295073 30886395

[56] Reinhold WC Sunshine M Liu H Varma S Kohn KW Morris J. CellMiner: A Web-Based Suite of Genomic and Pharmacologic Tools to Explore Transcript and Drug Patterns in the NCI-60 Cell Line Set. Cancer Res (2012) 72(14):3499–511. doi: 10.1158/0008-5472.Can-12-1370 PMC3399763 22802077

[57] Wu S Ma L Wu Y Zeng R Zhu X. Nudel Is Crucial for the WAVE Complex Assembly *In Vivo* by Selectively Promoting Subcomplex Stability and Formation Through Direct Interactions. Cell Res (2012) 22(8):1270. doi: 10.1038/cr.2012.47 22453242 PMC3411165

[58] Cohen J Raviv S Adir O Padmanabhan K Soffer A Luxenburg C. The Wave Complex Controls Epidermal Morphogenesis and Proliferation by Suppressing Wnt-Sox9 Signaling. J Cell Biol (2019) 218(4):1390–406. doi: 10.1083/jcb.201807216 PMC6446834 30867227

[59] Yao Q Tu C Lu D Zou Y Liu H Zhang S. Clinicopathological Significance of the microRNA-146a/WASP-Family Verprolin-Homologous Protein-2 Axis in Gastric Cancer. Cancer Sci (2017) 108(7):1285–92. doi: 10.1111/cas.13254 PMC5497796 28387985

[60] Wang J Feng Y Chen X Du Z Jiang S Ma S. SH3BP1-Induced Rac-Wave2 Pathway Activation Regulates Cervical Cancer Cell Migration, Invasion, and Chemoresistance to Cisplatin. J Cell Biochem (2018) 119(2):1733–45. doi: 10.1002/jcb.26334 28786507

[61] Takahashi K Suzuki K. WAVE2, N-WASP, and Mena Facilitate Cell Invasion *via* phosphatidylinositol 3-kinase-dependent local accumulation of actin filaments. J Cell Biochem (2011) 112(11):3421–9. doi: 10.1002/jcb.23276 21769917

