# Supplementary material for: WASF2 Serves as a Potential Biomarker and Therapeutic Target in Ovarian Cancer: A Pan-Cancer Analysis
Source: Front Oncol. 2022 Mar 14;12:840038. doi: 10.3389/fonc.2022.840038 (PMC8964075; doi:10.3389/fonc.2022.840038)
Supplement: Supplementary file 1 [file DataSheet_1.docx]

**WASF2 serves as a potential biomarker and therapeutic target in ovarian cancer: a pan-cancer analysis**

**Supplementary materials** **and methods**

***Patients and samples***

40 patients with ovarian cancer were enrolled in this study. All patients presented high level of Carbohydrate antigen 125 (CA-125) and didn't treat with chemotherapy or radiotherapy before surgery. Patients with stage III and stage IV received chemotherapy with carboplatin and gemcitabine after surgery. Matched 40 normal ovarian tissues from non-tumor ovarian lesions. Clinicalpathological characters were collected from medical records, including age, menopause, tumor type, FIGO stages and tumor grade.

***Immunohistochemistry***

The pathological tissue wax block was selected from the Department of Pathology of Jinan University. These tissues were fixed in 4% paraformaldehyde for 24–36 h following a standard protocol, then dehydrated and embedded in paraffin. Tissue sections (5-μm thickness) were mounted on glass slides (CITOTEST, China). Rabbit anti-WASF2 antibodies (1:200; cat.no. 3659, RRID: AB_2216981) were used as primary antibodies, and anti-rabbit IgG (1:1000; Abcam) used as a secondary antibody. The reaction was revealed using Novolink Polymer (Leica Mycrosystems, Newcastle upon Tyne, UK) followed by diaminobenzidine (DAB, Dako, Carpinteria, CA, USA) as chromogen. Sections were then counterstained with Mayer's haematoxylin. The results of the statistical analysis of immunohistochemistry were expressed by relative optical density (IOD). The median of IOD was used as the cut-off point for grouping the patients into high or low level of expression groups.

***Cell Line and Culture Conditions***

SKOV3 cells originating from human ovarian carcinoma tissues were obtained from ATCC (USA). Cells were cultured in Dulbeccos modified Eagles medium (DMEM, Invitrogen) with 10% foetal bovine serum (FBS, Gibco) at 37°C with 5% CO2.

***Transfection with small interfering (si) RNA***

siRNAs targeting WASF2 and control siRNA were synthesized and purified by Heyuan Biotechnology Co., Ltd. (Shanghai, China). Transfection was performed using Lipofectamine^®^ 3000 Transfection reagent (Invitrogen; Thermo Fisher Scientific, Inc.) following the manufacturer's protocols. Final concentration of siRNAs was 100 nM and cells were harvested 72 h following transfection.

***Western blot analysis***

Western blot analysis was performed following standard procedures. Total protein was extracted from SKOV3 cells using RIPA lysis buffer containing a protease inhibitors (BiYunTian, Shanghai, China), and protein concentrations were detected using the BCA protein assay kit (Thermo Fisher Scientific, Inc.). Total protein (30 µg/lane) was loaded into each well by SDS-PAGE using a 10% gel and then transferred to a polyvinylidene fluoride (PVDF) membrane. The membrane was then blocked with 5% nonfat dry milk for 1 hour at room temperature. Following overnight incubation at 4°C with rabbit mAb to WASF2 (1:1,000; cat.no. 3659, RRID: AB_2216981). Subsequently, the membrane was incubated with the corresponding anti-rabbit secondary antibody (1:5,000; cat. no. ab6721; Abcam) for 2 hours at room temperature and detected by chemiluminescence. β-actin(1:1,000; cat. no. ab8226; Abcam) was used as a loading control.

***Cell proliferation assay***

Cell proliferation was performed using Cell Counting Kit-8 (CCK8) (KeyGEN, Nanjing, China). Firstly, cultured cells were seeded into 96-well plates (1000 per well). Then, 10 μl CCK-8 solutions were added into each well at 24, 48, and 72 hours. Cells were incubated at 37℃ for 2h. The absorbance of each sample was measured with a microplate reader at a wavelength of 450nm.

***Actine staining***

Actine staining was performed using phalloidin coupled to fluorescein-isothyo-cyanate. After 48h of incubation, the cells on specimens were washed with PBS and then fixed with 4% formaldehyde for 15 min. Next, the cells were washed in PBS, dehydrated with acetone, permeated with 0.1% Triton X-100 in PBS, and rinsed again in PBS. Factin was stained using fluorescein (FITC) phalloidin (1:1000, Thermo Fisher Scientific, F432) at room temperature. After 2 hours, the cells were washed several times with PBS to remove unbound phalloidin conjugate. Nuclear stain DAPI was used to label the nuclei. Images were obtained by a fluorescence microscope.

***Wound healing assay***

A total of 3×10^5^ cells per well were seeded in 6-well plates, then transfected with WASF2 siRNA following overnight incubation. When cells reached 90% confluence, the cell monolayer was scratched with a sterile pipette tip and cells were cultured in DMEM for 12 h. Photographs were taken at 0, 6 and 12 h along the scrape line using a light microscope. Migration images were captured by an inverted microscope and the relative percentage of wound closure was determined by comparison with control cells.

***Invasion assay***

Invasion analysis was conducted by Transwell inserts with polycarbonate membranes (8.0 μm pore size) with Matrixgel (BD). SKOV3 cells (4×10^4^ cells/well) were seeded into the upper chamber and culture medium containing 20% FBS was added in the lower chamber. After 24h of incubation, non-migrated cells in the upper chamber were removed with cotton swabs. The migrated cells were fixed with 4% paraformaldehyde , stained with crystal violet and counted.

**Supplementary table**

Suppl Table 1. The protein expression levels of WASF2 were associated with clinicalpathological characters.

| Clinicalpathological characters | N | WASF2 | | χ2 | *P* value |
| --- | --- | --- | --- | --- | --- |
|  |  | Low expression | High expression |  |  |
| Age (years) |  |  |  | 0.47 | 0.538 |
| ≤54 | 18 | 10 | 7 |  |  |
| >54 | 22 | 11 | 12 |  |  |
| Tumor type |  |  |  | 3.09 | 0.105 |
| Type I | 14 | 10 | 4 |  |  |
| Type II | 26 | 11 | 15 |  |  |
| Menopause |  |  |  | 0.07 | 0.796 |
| Positive | 16 | 8 | 8 |  |  |
| Negative | 24 | 13 | 11 |  |  |
| FIGO stages |  |  |  | 7.28 | 0.010^a^ |
| I–II | 15 | 12 | 3 |  |  |
| III–IV | 25 | 9 | 16 |  |  |
| Tumor grade |  |  |  | 0.99 | 0.608 |
| 1 | 12 | 5 | 7 |  |  |
| 2 | 15 | 8 | 7 |  |  |
| 3 | 13 | 8 | 5 |  |  |

^a^P<0.05; FIGO, International Federation of Gynecology and Obstetrics.

**Supplementary figure legends**

Supplement Figure 1: WASF2 is expressed in ovarian cancer and normal cells. (A) WASF2 is highly expressed in ovarian cancer. (B) The expression level of WASF2 in different stage of ovarian cancer. (C) The expression data of WASF2 in different normal cells.

Supplement Figure 2: The statistical chart after using the CIBERSORT method shows the proportion difference of immune cells between WASF2 high and low expression groups in ovarian cancer. Red represents the high WASF2 expression group, yellow represents the low WASF2 expression group.

Supplement Figure 3: Correlation between the expression of WASF2 and immune cells infiltration in pan-cancer analysis. Red indicates a correlation coefficient > 0, whereas blue indicates a correlation coefficient < 0.

Supplement Figure4: Correlation analysis of WASF2 expression with ESTIMATE scores. Red indicates a correlation coefficient > 0, whereas blue indicates a correlation coefficient < 0.

Supplement Figure5: WASF2 expression is correlated with common tumor-related regulatory genes. (A) The correlation between WASF2 and autophagy gene. (B) The correlation between WASF2 and DNA repair gene. (C) The correlation between WASF2 and ferroptosis gene. (D) The correlation between WASF2 and hypoxia gene. (E) The correlation between WASF2 and pyroptosis gene. (F) The correlation between WASF2 and TGF-β signaling gene. **P*< 0.05; ***P*< 0.01; ****P*< 0.001.

Supplement Figure 6: KEGG pathway analysis of WASF2 in ovarian cancer. Curves of different colors show different pathways regulated in ovarian cancer. Peaks on the upward curve indicate positive regulation and peaks on the downward curve indicate negative regulation.
