# Supplementary figures and images for: WASF2 Serves as a Potential Biomarker and Therapeutic Target in Ovarian Cancer: A Pan-Cancer Analysis
Source: Front Oncol. 2022 Mar 14;12:840038. doi: 10.3389/fonc.2022.840038 (PMC8964075; doi:10.3389/fonc.2022.840038)

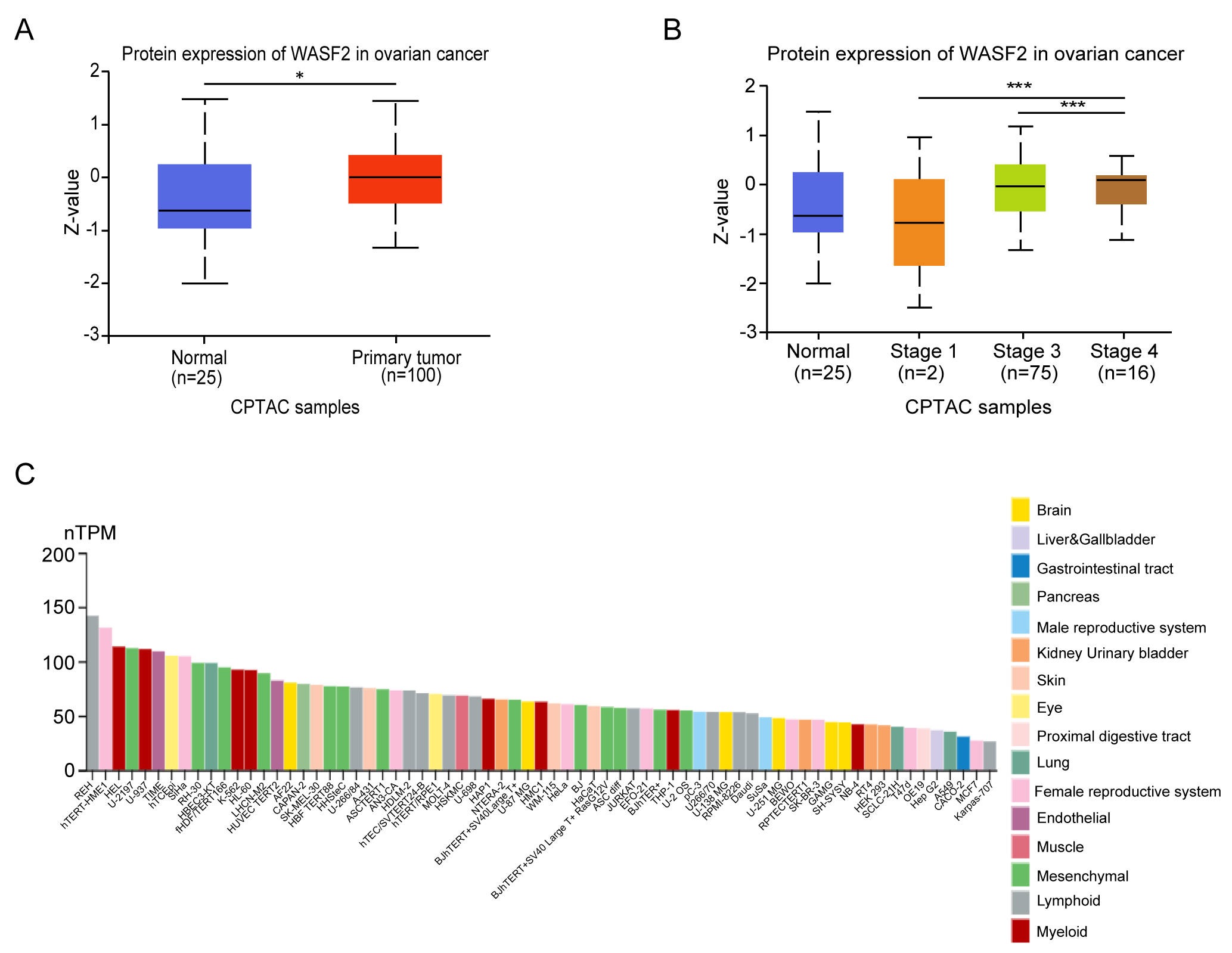

Supplement: Supplementary file 2 [file Image_1.tif]

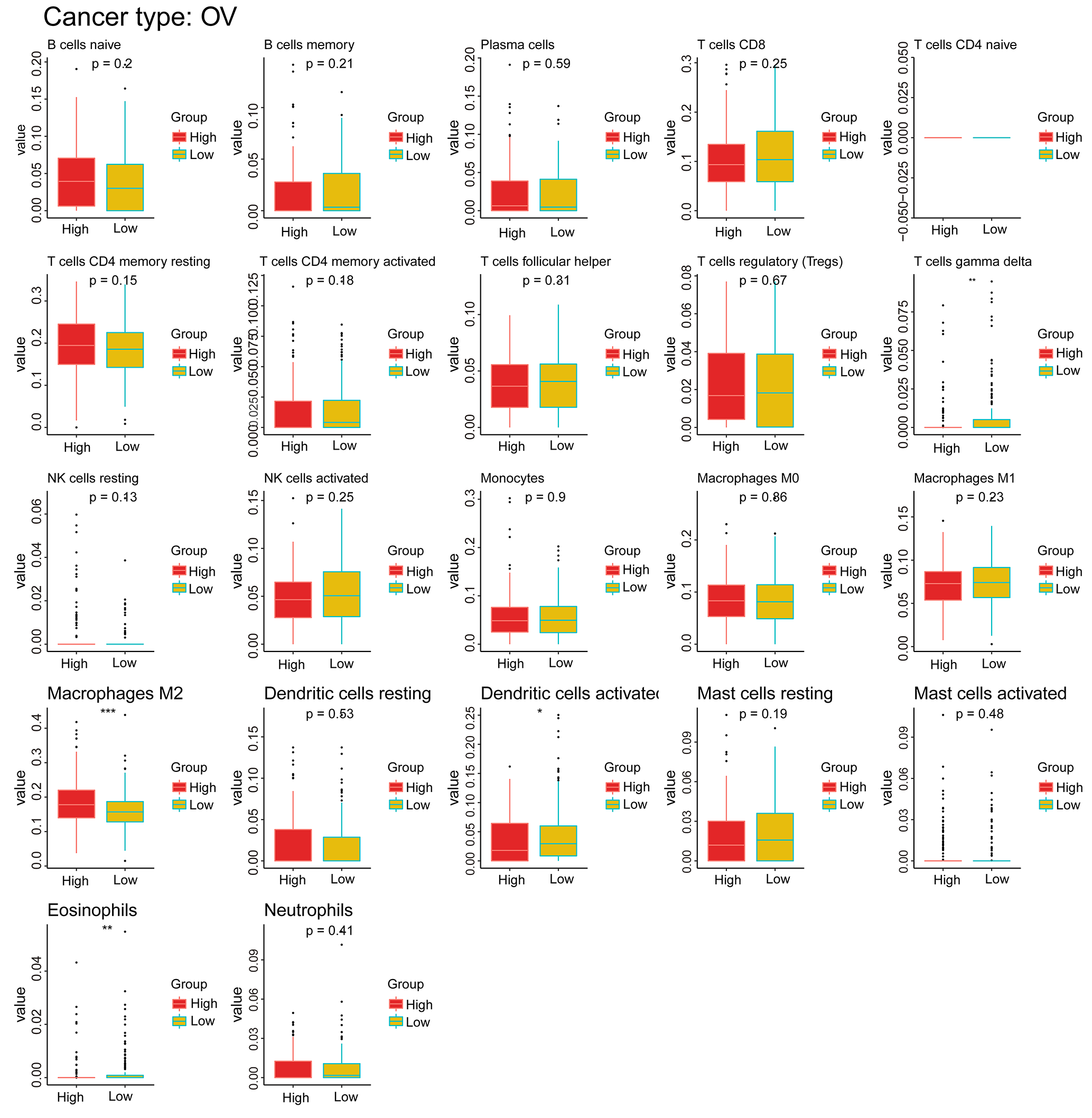

Supplement: Supplementary file 3 [file Image_2.tif]

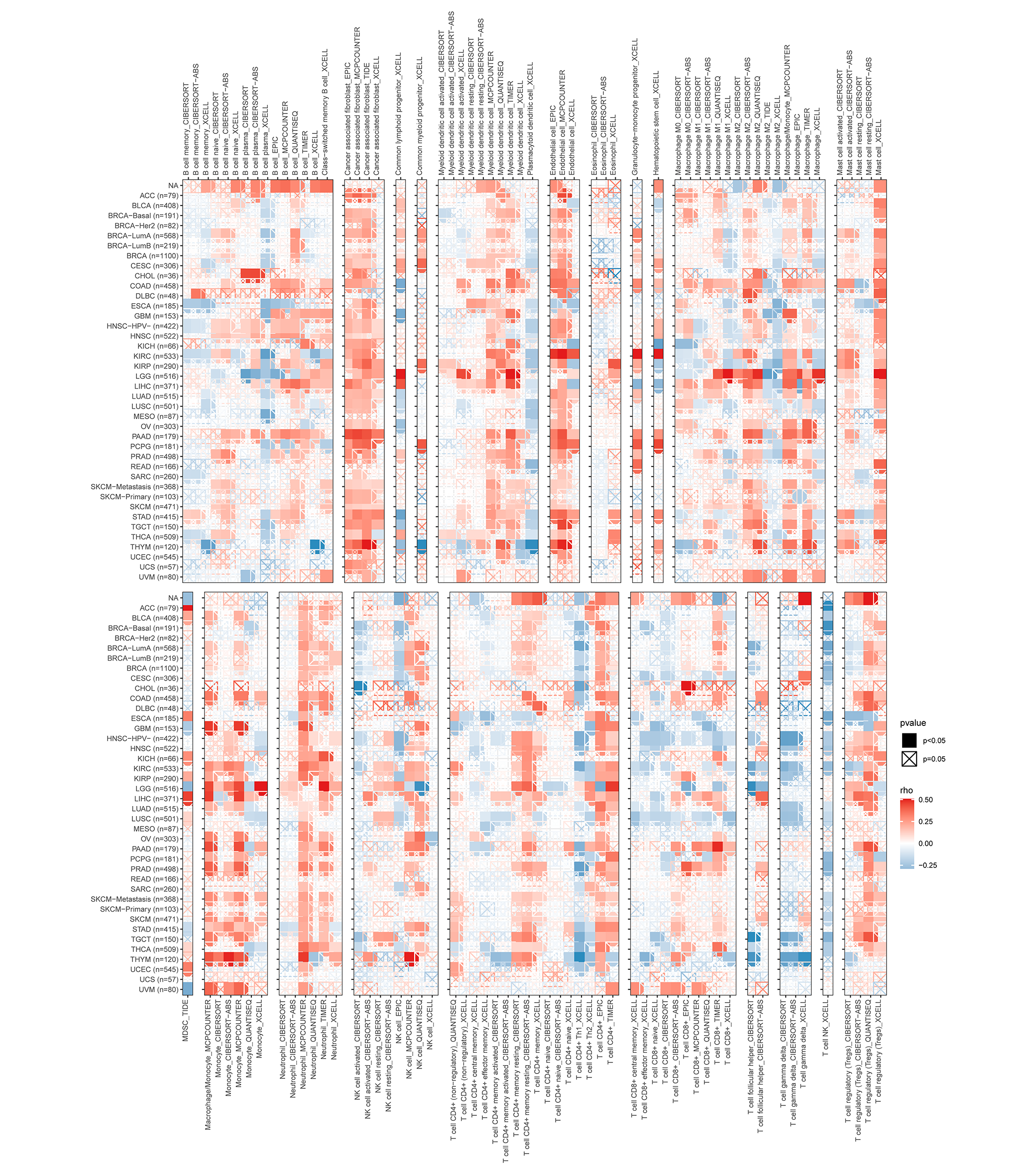

Supplement: Supplementary file 4 [file Image_3.tif]

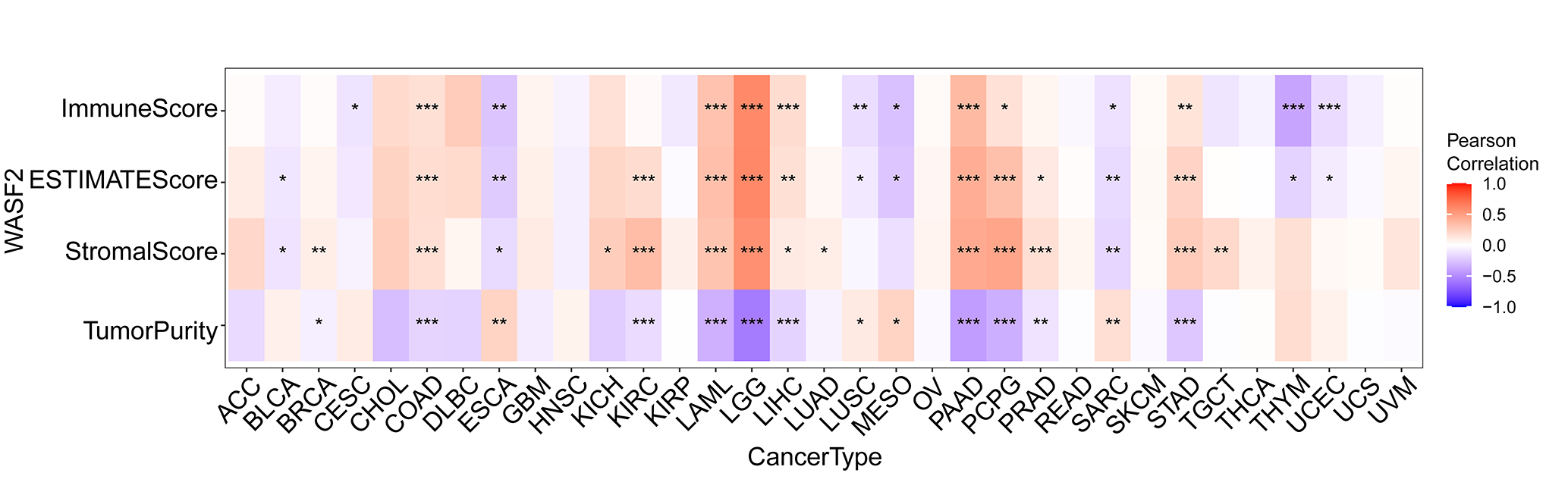

Supplement: Supplementary file 5 [file Image_4.tif]

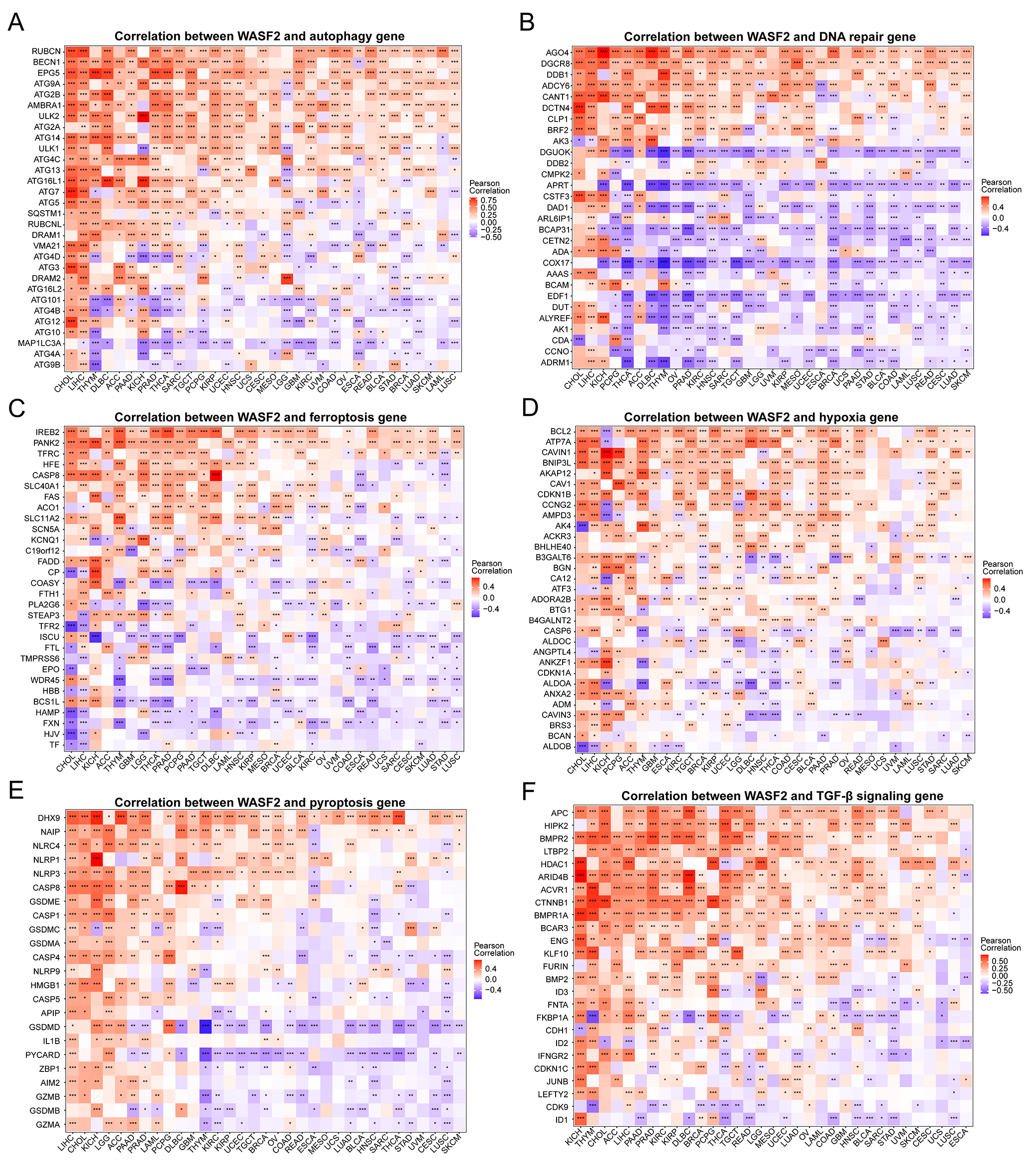

Supplement: Supplementary file 6 [file Image_5.tif]

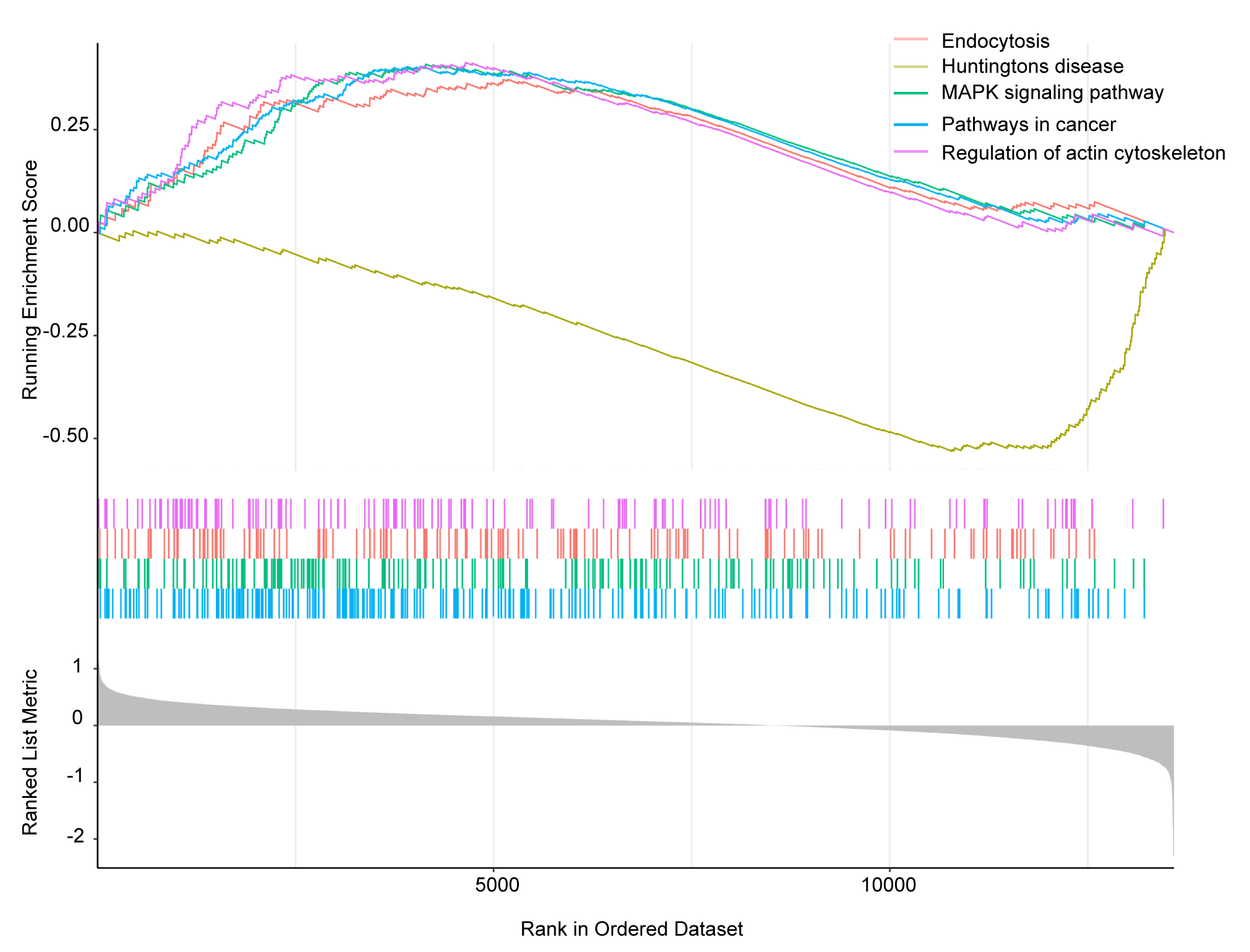

Supplement: Supplementary file 7 [file Image_6.tif]
